# Mosaic loss of Y chromosome and mortality after coronary angiography

**DOI:** 10.1093/eurheartj/ehaf035

**Published:** 2025-02-12

**Authors:** Michael Weyrich, Stephen Zewinger, Tamim Sarakpi, Tina Rasper, Marcus E Kleber, Sebastian Cremer, Lukas Zanders, Fenja Fleck, Agneta Siegbahn, Lars Wallentin, Wesley Tyler Abplanalp, Linda Nerbas, Sandra Fay, Aaron L Eberle, Stefanie Dimmeler, Winfried März, Thimoteus Speer, Andreas M Zeiher

**Affiliations:** Department of Internal Medicine 4, Nephrology, Goethe University Frankfurt, University Hospital, Theodor-Stern-Kai 7, Frankfurt 60590, Germany; Else Kroener-Fresenius Center for Nephrological Research, Goethe University Frankfurt, Theodor-Stern-Kai 7, Frankfurt 60590, Germany; Department of Internal Medicine 4, Nephrology, Goethe University Frankfurt, University Hospital, Theodor-Stern-Kai 7, Frankfurt 60590, Germany; Hôpital Robert Schumann, Hôpital Kirchberg, Luxemburg, Luxemburg; Medical Faculty, Saarland University, Homburg/Saar, Germany; Department of Internal Medicine 4, Nephrology, Goethe University Frankfurt, University Hospital, Theodor-Stern-Kai 7, Frankfurt 60590, Germany; Else Kroener-Fresenius Center for Nephrological Research, Goethe University Frankfurt, Theodor-Stern-Kai 7, Frankfurt 60590, Germany; Institute for Cardiovascular Regeneration, Goethe University Frankfurt, Frankfurt, Germany; Vth Department of Medicine, University of Heidelberg, University Medical Center, Medical Faculty Mannheim, Mannheim, Germany; SYNLAB MVZ Humangenetik Mannheim GmbH, Mannheim, Germany; Department of Medicine, Cardiology, Goethe University Frankfurt, University Hospital, Frankfurt, Germany; German Center for Cardiovascular Research DZHK, Partner Site Frankfurt, Theodor-Stern-Kai 7, Frankfurt 60590, Germany; Department of Medicine, Cardiology, Goethe University Frankfurt, University Hospital, Frankfurt, Germany; German Center for Cardiovascular Research DZHK, Partner Site Frankfurt, Theodor-Stern-Kai 7, Frankfurt 60590, Germany; Institute for Cardiovascular Regeneration, Goethe University Frankfurt, Frankfurt, Germany; Department of Medical Sciences, Clinical Chemistry, Uppsala University, Uppsala, Sweden; Uppsala Clinical Research Center, Uppsala University, Uppsala, Sweden; Science for Life Laboratory, Uppsala University, Uppsala, Sweden; Uppsala Clinical Research Center, Uppsala University, Uppsala, Sweden; Department of Medical Sciences, Cardiology, Uppsala University, Uppsala, Sweden; Institute for Cardiovascular Regeneration, Goethe University Frankfurt, Frankfurt, Germany; German Center for Cardiovascular Research DZHK, Partner Site Frankfurt, Theodor-Stern-Kai 7, Frankfurt 60590, Germany; Department of Internal Medicine 4, Nephrology, Goethe University Frankfurt, University Hospital, Theodor-Stern-Kai 7, Frankfurt 60590, Germany; Else Kroener-Fresenius Center for Nephrological Research, Goethe University Frankfurt, Theodor-Stern-Kai 7, Frankfurt 60590, Germany; Department of Internal Medicine 4, Nephrology, Goethe University Frankfurt, University Hospital, Theodor-Stern-Kai 7, Frankfurt 60590, Germany; Else Kroener-Fresenius Center for Nephrological Research, Goethe University Frankfurt, Theodor-Stern-Kai 7, Frankfurt 60590, Germany; Department of Internal Medicine 4, Nephrology, Goethe University Frankfurt, University Hospital, Theodor-Stern-Kai 7, Frankfurt 60590, Germany; Else Kroener-Fresenius Center for Nephrological Research, Goethe University Frankfurt, Theodor-Stern-Kai 7, Frankfurt 60590, Germany; Institute for Cardiovascular Regeneration, Goethe University Frankfurt, Frankfurt, Germany; German Center for Cardiovascular Research DZHK, Partner Site Frankfurt, Theodor-Stern-Kai 7, Frankfurt 60590, Germany; Vth Department of Medicine, University of Heidelberg, University Medical Center, Medical Faculty Mannheim, Mannheim, Germany; Clinical Institute of Medical and Chemical Laboratory Diagnostics, Medical University of Graz, Graz, Austria; SYNLAB Holding Deutschland GmbH, SYNLAB Academy, Mannheim, Germany; Department of Internal Medicine 4, Nephrology, Goethe University Frankfurt, University Hospital, Theodor-Stern-Kai 7, Frankfurt 60590, Germany; Else Kroener-Fresenius Center for Nephrological Research, Goethe University Frankfurt, Theodor-Stern-Kai 7, Frankfurt 60590, Germany; German Center for Cardiovascular Research DZHK, Partner Site Frankfurt, Theodor-Stern-Kai 7, Frankfurt 60590, Germany; Institute for Cardiovascular Regeneration, Goethe University Frankfurt, Frankfurt, Germany; German Center for Cardiovascular Research DZHK, Partner Site Frankfurt, Theodor-Stern-Kai 7, Frankfurt 60590, Germany

**Keywords:** Coronary artery disease, Loss of Y chromosome, Fibrosis

## Abstract

**Background and Aims:**

Acquired somatic mutations emerged as important drivers of adverse cardiovascular disease outcomes. Recently, mosaic loss of Y chromosome (LOY) in haematopoietic cells was identified to induce diffuse cardiac fibrosis in male mice. The aim of the present study was to determine the association between LOY and cardiovascular mortality in patients undergoing coronary angiography.

**Methods:**

LOY was quantified in 1698 male participants of the LURIC study, who underwent coronary angiography, and its association with all-cause and cardiovascular mortality was determined. Furthermore, the interaction between LOY and inherited genetic susceptibility for cardiac fibrosis was assessed.

**Results:**

The frequency of LOY steeply increased in male participants of LURIC at the age of 60 years. Loss of Y chromosome > 17% was associated with significantly higher all-cause [hazard ratio (HR) 1.41, 95% confidence interval (CI) 1.09–1.82] and cardiovascular mortality (HR 1.49, 95% CI 1.09–2.03), which was driven by a higher risk for fatal myocardial infarction (HR 2.65, 95% CI 1.46–4.81). Loss of Y chromosome > 17% was associated with a profibrotic and proinflammatory plasma protein expression profile as characterized by higher plasma levels of osteoprotegerin, matrix metalloproteinase-12, growth differentiation factor 15, heparin-binding EGF-like growth factor, and resistin. Genetic predisposition for lower myocardial fibrosis attenuated the association between LOY and cardiovascular mortality. Genome-wide methylation analyses identified differential methylation in 298 genes including *ACTB*, *RPS5*, *WDR1*, *CD151*, and *ARAP1*. Single-cell RNA sequencing further confirmed differential gene expression of 37 of these genes in LOY in peripheral blood mononuclear cells comprising a set of fibrosis-regulating genes including *RPS5*. *RPS5* silencing in macrophages induced a paracrine induction of collagen expression in cardiac fibroblasts documenting a functional role *in vitro*.

**Conclusions:**

LOY represents an important independent risk factor for cardiovascular mortality in male patients with coronary artery disease. Targeting LOY may represent a sex-specific personalized medicine approach.

## Structured Graphical Abstract

**Structured graphical abstract:**
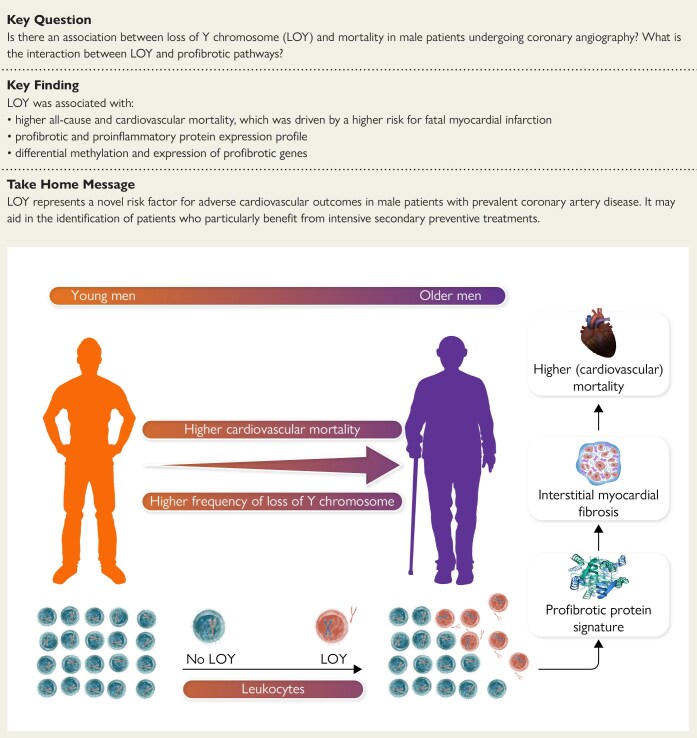
Frequency of loss of Y chromosome (LOY) in leukocytes increases with age, associates with a profibrotic protein signature, interstitial myocardial fibrosis, and leads to higher cardiovascular mortality.

**See the editorial comment for this article ‘Loss of the Y chromosome in coronary artery disease', by S. Sano and K. Walsh, https://doi.org/10.1093/eurheartj/ehaf072.**

## Introduction

Cardiovascular diseases (CVDs) represent a significant global health burden, contributing to substantial morbidity and mortality. Given the continuous growth of the elderly population, ischaemic heart disease is anticipated to persist as the primary cause of death in 2040.^1^

Men have been shown to exhibit distinct patterns in both prevalence, manifestations, and outcome of CVDs compared with women.^2^ Importantly, it has been shown that modifiable cardiovascular risk factors (i.e. body mass index, systolic blood pressure, non-HDL cholesterol, current smoking, and diabetes) contribute only 52.6% to the 10-year incidence of CVD in men.^3^ This indicates that approximately 50% of the cardiovascular risk cannot be explained by classical cardiovascular risk factors.

Recent research has unveiled intriguing associations between genetic factors and cardiovascular health beyond traditional risk factors.^4,5^ Among those, the loss of Y chromosome (LOY) has garnered significant attention for its potential role in predisposing individuals, particularly men, to CVDs.^6,7^ Population-wide studies have demonstrated that LOY, characterized by the mosaic LOY in a subset of leukocytes, represents the most frequent acquired age-related somatic mutation, which can be detected in >20% of males above 60 years of age.^7^ Besides inherited, genetic risk factors that predispose to LOY,^7^ lifestyle-associated factors such as smoking have been documented to be associated with a higher frequency of LOY.^8^ Subsequently, LOY leads to alterations in distinct autosomal genes in leukocytes, which favours clonal expansion of these cells.^7^

It has been shown that LOY in haematopoietic cells promotes cardiac fibrosis and deteriorates the outcome of mice subjected to experimental transverse aortic constriction as a model for pressure overload.^9^ Loss of Y chromosome was associated with lower left ventricular function, accumulation of fibroblasts in the myocardium, and increased diffuse cardiac fibrosis. Furthermore, in the UK Biobank, a higher fraction of LOY was associated with higher all-cause and cardiovascular mortality.^9^ Recently, we have documented that the presence of LOY in monocytes correlated with significantly higher mortality in patients with severe degenerative aortic valve stenosis undergoing transcatheter aortic valve replacement^10^ and a higher risk for heart failure events in patients with chronic kidney disease.^11^ Furthermore, single-cell RNA sequencing (scRNAseq) revealed a profibrotic gene signature in monocytes from patients with LOY promoting transforming growth factor-β (TGF-β)-dependent signalling pathways in these cells.^10^

The aim of the present study was to quantify LOY in a well-characterized cohort of patients undergoing coronary angiography and to evaluate LOY as a risk factor for adverse outcomes in males at high risk of coronary artery disease (CAD) during long-term follow-up.

## Methods

### LURIC study

The Ludwigshafen Risk and Cardiovascular Health (LURIC) study is an observational study that enrolled 3316 participants undergoing coronary angiography recruited in Germany between 1997 and 2000 with a median follow-up of 9.9 years.^12–14^ Participants with acute illnesses other than acute coronary syndromes, such as malignancies or other chronic non-cardiac conditions within the past 5 years, were ineligible for participation *a priori*. Of the participants, 2310 were males, and 1698 male participants with available DNA samples for quantification of LOY were included in the present study (see Supplementary data online, *Figure S1A*). Data regarding mortality during the follow-up period were sourced from local public health records. Cardiovascular mortality was specifically defined as death resulting from fatal myocardial infarction, sudden cardiac events, post-interventional cardiac-related deaths, strokes, and other deaths attributed to CVD. Blood samples, inclusive of baseline clinical chemistry parameters, were collected on the day of coronary angiography preceding percutaneous coronary intervention, if clinically indicated. The study adhered to the principles of the Declaration of Helsinki and was approved by the ethics committee of the Landesärztekammer Rheinland-Pfalz [approval number 837.255.97 (1394)]. Written informed consent was obtained from all patients. No patients were lost to follow-up for assessment of mortality causes.

### Quantification of loss of Y chromosome

Loss of Y chromosome was quantified using a previously validated polymerase chain reaction (PCR) technique as outlined in detail elsewhere.^10^ In brief, the relative number of X and Y chromosomes within DNA samples was determined employing a TaqMan-based method targeting a 6 bp sequence difference between the *AMELX* and *AMELY* genes, utilizing the same primer pair. Consequently, this approach is relatively unbiased concerning primer characteristics. A total of 115 ng of DNA was combined with PROBE PCR master mix (QIAcuity Probe PCR Kit, Qiagen, Germany) containing FastDigest HindIII enzyme (Thermo Fisher, USA) and TaqMan Primers (Thermo Fisher, USA). Following digestion (10 min, room temperature), the reaction mix was transferred to a 26k 24-well Nanoplate and loaded into a QIAcuity ONE instrument. Polymerase chain reaction amplification was conducted according to the manufacturer’s protocol: initial denaturation at 95°C for 2 min, followed by two-step cycling (40 cycles) at 95°C for 15 s and 60°C for 30 s. A 6 nt deletion is present in the X-specific amelogenin gene (B37/hg19 genome locations: chrX:11315039 and chrY:6737949–6737954). The VIC dye probe detects X chromosome sequences, while the FAM dye probe, inclusive of the 6 nt deletion, detects Y chromosome sequences [sequence: GTGTTGATTCTTTATCCCAGATG[-/AAGTGG]TTTCTCAAGTGGTCCTGATTTT (VIC/FAM)]. Endpoint fluorescence intensity of the partitions was separately assessed for FAM (*AMELY*) and VIC (*AMELX*) to determine the presence or absence of the respective targets. The absolute concentration of the targets was computed using the QIAcuity One Software suite (Qiagen, Germany) based on the ratio of positive and negative partitions, thereby deriving the *AMELY*/*AMELX* ratio. The extent of LOY was defined as the percentage of cells with LOY derived from this ratio.

### OLINK cardiovascular proteomics panel

Normalized protein expression (NPX) of 80 CVD-related proteins was quantified using the Proseek Multiplex CVD I array according to the manufacturer’s protocol.

### Genotyping in LURIC

In the LURIC study, genotyping was carried out utilizing the Affymetrix Human SNP Array 6.0, as detailed previously.^12^ Subsequent imputation to the Phase 1 1000 Genomes panel (March 2012 release) was performed using IMPUTE2.

### Weighted polygenic risk score for myocardial fibrosis

We calculated a previously reported weighted polygenic risk score for myocardial fibrosis.^15^ The score is based on 11 single nucleotide polymorphisms (SNPs) previously shown to be associated with T1 time derived from cardiac magnetic resonance imaging data from UK Biobank at genome-wide significance. The score includes the following variants: rs2627230, rs9457699, rs1576900, rs6120777, rs115740542, rs855791, rs55754224, rs365843, rs58774558, rs13097267, and rs199754787. The weighted polygenic risk score was built using a common approach^16^ by computing the sum of risk alleles weighted by the risk allele effect sizes. Details on the wPRS have been previously published.^15^ Briefly, genome-wide significant variants (*P* < 5 × 10^−8^) were selected and pruned (LD threshold *r*^2^ < .01 within a 1000 kb window) using 1000G multi-ancestry or European Phase 3 LD data, based on ancestral composition of available summary statistics to identify variants associated with each CV risk factors. Next, external summary statistics were harmonized with MRI T1 time summary statistics by transforming genomic coordinates to Genome Reference Consortium Human build 37, standardizing strand orientation, and aligning effect alleles and effect estimates. Variants with minor allele frequency < 1% or poor imputations quality (INFO score < .3) were excluded. Analyses using genetic risk factors were restricted to European samples. Analyses were adjusted for 20 genotyping principal components.

### Methylation analyses

DNA methylation of whole blood was quantified using the Illumina Infinium MethylationEPIC BeadChip following the manufacturer’s protocols. This array encompasses more than 850 000 methylation sites across nuclear DNA. Quality control procedures were enforced utilizing the CPACOR pipeline,^17^ which entailed excluding samples with a call rate of ≤.95 and those exhibiting sex discordance. CpGs positioned within close proximity (1–2 bp) to a genetic polymorphism prevalent in the European population with a frequency exceeding .01%, along with cross-reactive probes and probes displaying a detection *P*-value exceeding .05 in at least 1% of the samples, were eliminated using the rmSNPandCH function within the DMRcate package,^18^ followed by quantile normalization. Subsequently, a total of 795 619 autosomal and 18 138 X chromosomal CpGs from 2423 samples were retained for further analyses. For the analyses, beta values of normalized probe intensities were used. *P*-values were adjusted for multiple testing by using the Holm–Bonferroni method. Analyses were adjusted for age, composition of leukocytes, and Chip id, as well as 30 principal components as calculated from control–probe intensities. Results were visualized using a Circos plot.

### Single-cell RNA sequencing

We analysed the expression of all differentially methylated genes (*N* = 298) by using scRNAseq as previously published^10^ comprising pooled data from seven male patients with severe degenerative aortic stenosis. Loss of Y chromosome cells were defined as cell lacking expression of all Y chromosome-derived genes. Relative changes in transcription of Y harbouring and LOY peripheral blood mononuclear cells (PBMCs) were examined. *P*-values were adjusted for multiple testing by using the Bonferroni correction.

### Cell culture

Human cardiac fibroblasts (HCFs) were purchased from Promocell (494Z037.1) and cultured in fibroblast growth medium (Promocell, C-231130) with 1% penicillin/streptomycin (P/S) (Gibco) at 37°C, 5% CO_2_ according to the manufacturer’s protocol. THP-1 cells were purchased from German Collection of Microorganisms and Cell Cultures (ACC16) and cultured in RPMI1640 supplemented with 10% heat-inactivated FCS, 1% P/S and 10 mM HEPES at 5% CO_2_, 37°C with passaging every 2–3 days. THP-1 cells were seeded with 5*10^5^ cells/wells and differentiated into macrophages by incubation with 100 ng/mL phorbol 12-myristate-13-acetate (PMA, Sigma-Aldrich, 16561-29-8) for 48 h.

### Small interfering RNA transfection of phorbol 12-myristate-13-acetate-differentiated THP1-cells

Phorbol 12-myristate-13-acetate-differentiated THP1-cells were transfected with small interfering RNA (siRNA)-targeting human *RPS5* (Dharmacon, L-010498-00-0005), *ZNF138* (Dharmacon, L-021278-02-0005), or *EEF1D* (Dharmacon, L-011648-01-0005) by using siTran 2.0 transfection reagent (Origen, TT320001). For this purpose, cells were incubated with fresh medium 30 min before transfection and then transfected with 75 nM siRNA. Non-targeting control siRNA (Dharmacon, D-001320-10-05) was used as a negative control. Cells were incubated overnight, and the medium was replaced the next day. After 24 h, supernatant was collected and transfection efficiency was assessed by quantitative real-time PCR.

### Quantitative real-time PCR

RNA was isolated from PMA-differentiated THP-1 cells using RNeasy Plus Mini Kit (Qiagen, 74136) according to the manufacturer’s instructions. Afterwards, RNA was transcribed to cDNA using Random Hexamer Primer (Thermo Fisher Scientific, SO142) and M-MLV Reverse Transcriptase (200 U/µL, Invitrogen, 28-025-013). Quantitative real-time PCR was performed with Fast SYBR Green Master Mix (Thermo Fisher Scientific, 4385610) on a Viia 7 Real-Time PCR System (Thermo Fisher Scientific) with specific primers for *RPS5* (Merck, forward CTACGCCGAGTGACAGAGAC, reverse GCTCCACAATGGGACACTGA), *ZNF138* (Merck, forward GTGGAAAAGCCTTTCACCAATCC, reverse AAGGCTTTGCCACAGTGTGCAC), and *EEF1D* (Merck, forward GGATTGCCAGTCTGGAAGTGGA, reverse AGCTCTTCTCCAGCACGTTCAG). Gene expression was calculated with the 2^−deltaCT^ method using the housekeeping genes *RPLP0* and *GAPDH* to normalize the gene expression.

### Fibroblast stimulation

Fibroblasts were seeded in an 18-well plate (ibidi, 81817) and allowed to adhere overnight. On the following day, the medium was changed with a 1:1 ratio of fibroblast growth medium, 1% P/S, and the supernatants obtained from the siRNA transfected PMA-differentiated THP-1 cells. As positive and negative control, HCFs were stimulated using 50 ng/mL recombinant human (rh) TGF-ß1 (Peprotech, 200-21) in full medium or left unstimulated in full medium, respectively. Stimulation was performed for 72 h, after which the cells were fixed and stained for further analysis.

### Immunofluorescence

Stimulated fibroblasts were fixed with 4% paraformaldehyde (Thermo Scientific, 28906) for 10 min. Afterwards, they were washed with PBS, permeabilized with .1% Triton-X for 10 min, and then blocked with 5% donkey serum for 1 h. Thereafter, cells were incubated overnight with a COL1A1 (E8F4L) rabbit mAb (Cell Signaling 72026, 1:200) and Oregon Green 488 phalloidin (Invitrogen O7466, 1:400). The cells were then washed with PBS followed by incubation for 1 h with the secondary antibody (Donkey anti-Rabbit IgG (H + L) Highly Cross-Adsorbed Secondary Antibody, Alexa Fluor™ 647, Thermo-Fisher, A-31573, 1:200), and DAPI (Thermo-Fisher, 62248, 1:1000). Finally, they were covered with Fluoromount-G™ Mounting Medium (Thermo-Fisher, 00-4958-02).

### Confocal imaging

Images were obtained using a Zeiss LSM 980 laser scanning confocal microscope equipped with an Airyscan2 detector. Images were taken as Z-stacks (20 µm) with a 10× objective and a 2× zoom using Airyscan2 detector. Images were converted into max projections using ZEN software (Carl Zeiss Microscopy GmbH, Version 3.9.101.06000) and then analysed with ZEN. The area of collagen was measured and then normalized to the area of DAPI.

### Statistical analyses

Continuous variables are reported as median (25%–75% percentile). Categorical variables are presented as counts and percentages. Statistical differences among continuous variables were evaluated using the Mann–Whitney *U* test for non-normally distributed variables, alongside the *χ*^2^ test for categorical variables. All observations were included in the current analyses.

Patients were divided into two groups at LOY of 17%, a cut-off for LOY recently established using the Youden index based on the area under the curve from receiver-operating curve analyses in patients undergoing transcatheter aortic valve replacement for severe degenerative aortic valve stenosis.^10^ To rule out that the association between LOY and the distinct outcomes does not depend on a specific LOY cut-off, LOY was also included as a continuous variable (transformed as *Z*-score) in the respective models. The assumption of linearity for quantitative predictors was evaluated through visual examination of the plots depicting residuals against fitted values.

The associations between LOY and age and LOY and all-cause mortality were visualized by restricted cubic spline plots, with three knots placed at the 10th, 50th, and 90th percentiles of age or LOY, respectively.

Cox proportional hazard models were built to examine the relationship between LOY and the mortality. We report results of two adjusted models: (1) adjusted for age, age^2^, diabetes, hypertension, smoking, and high-sensitivity C-reactive protein (hsCRP) as derived from a directed acyclic graph as shown in Supplementary data online, *Figure S1B* as minimal sufficient set of variables and (2) adjusted for age, age^2^, smoking status, body mass index, LDL cholesterol (LDL-C), hsCRP, troponin T, diabetes, hypertension, myocardial infarction, and CAD. Cumulative survival function was depicted by survival plots, where covariates were set to their mean. The proportional hazard assumption was assessed in STATA using the ‘estat phtest’ command and by plotting scaled Schoenfeld residuals. The linearity assumption for quantitative predictors was evaluated by plotting Martingale residuals against the quantitative predictors using the ‘predict mgale’ command within the STATA ‘postestimation’ command. To examine the association between LOY and all-cause mortality according to baseline characteristics, we have included a first-order interaction term between LOY and the respective baseline variable in the respective Cox regression models. The association between LOY and cardiovascular mortality (competing event: non-cardiovascular mortality), mortality due to fatal myocardial infarction (competing events: other cardiovascular mortality and non-cardiovascular mortality), and cancer mortality (competing events: non-cancer mortality) was determined by competing risk regression using Fine–Gray models implemented in the stata command ‘stcrreg’. Analyses were adjusted as described above. Subhazard ratios (SHR) and 95% confidence intervals (CIs) were reported, and results were depicted by cumulative incidence curves.

We built linear regression models to determine the association between baseline variables and LOY. Multivariate adjusted least square means of LOY are reported. Analyses were adjusted for age, smoking status, body mass index, LDL-C, hsCRP, troponin T, diabetes, hypertension, myocardial infarction, and CAD.

Linear regression models were built to determine the association between LOY and OLINK protein array-derived normalized plasma protein expression (NPX) levels. Analyses were adjusted for age, smoking status, body mass index, LDL-C, hsCRP, troponin T, diabetes, hypertension, myocardial infarction, CAD, and number of NPX values below detection across all proteins. To account for multiple testing, *P*-values were calculated at FDR of .05 (*P*-value*rank/number of comparisons, i.e. *N* = 80).

In general, a two-sided *P* < .05 was considered statistically significant. Analyses were performed using SPSS version 21.0 and STATA IC 15 with the packages postrcspline, estat phtest, postestimation, estat gof, and stcrreg. Directed acyclic graph was generated using DAGitty. Circos plot was used to visualize genome-wide methylation and scRNAseq data.^19^ ScRNAseq analysis was performed by using R (version 4.0.3) and the analysis tool Seurat (v4.0.3). Differential gene expression was analysed using the ‘FindMarkers’ function from the Seurat package. The Wilcoxon rank sum test, followed by Bonferroni correction, was applied for significance testing. Results were considered significant if the adjusted *P*-value was less than .05.

## Results

### Loss of Y chromosome and all-cause mortality

**Figure 1:**
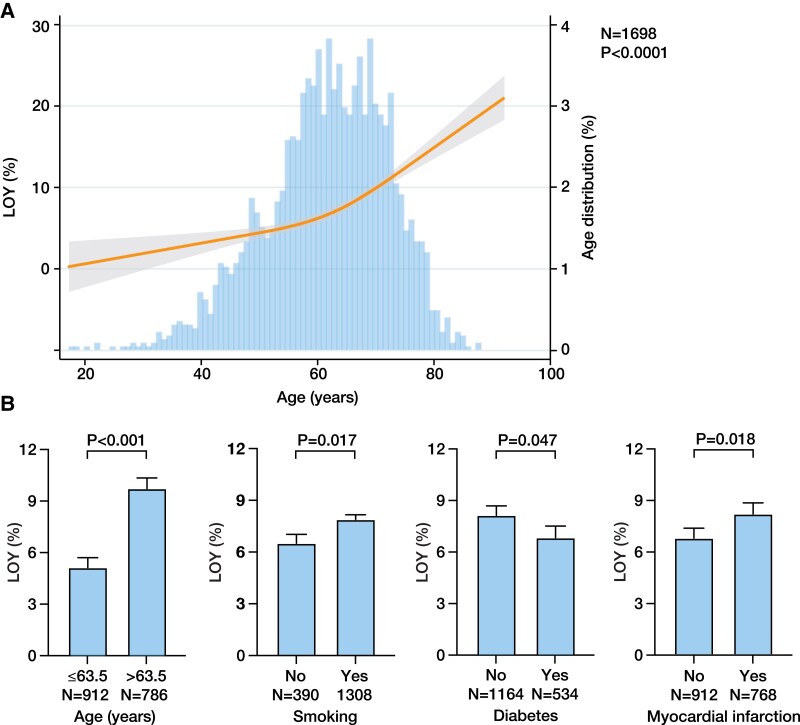
Association between loss of Y chromosome and baseline characteristics. (*A*) Restricted cubic spline plot of the association between age and loss of Y chromosome. The line indicates loss of Y chromosome (%) with the 95% confidence interval (grey-shaded area). Histogram shows the age distribution (right *Y* axis). (*B*) Multivariate adjusted least square means and the corresponding 95% confidence interval of loss of Y chromosome as determined by using linear regression models according to age (divided at the median), smoking status, diabetes, and myocardial infarction (all analyses are shown in Supplementary data online, *Table S1*). Analyses are adjusted for age, testosterone, triglycerides, LDL-C, glycated haemoglobin, Friesinger score, troponin T, diabetes, hypertension, coronary artery disease, smoking, myocardial infarction, lipid-lowering therapy, smoking, and high-sensitivity C-reactive protein

Loss of Y chromosome was quantified in 1698 male participants of the LURIC study. While the frequency of LOY was low in patients younger than 60 years, it steeply increased at the age of 60 years (*Figure 1A*; *P* < .0001 for the association between LOY and age). We first assessed the association between the extent of LOY and pre-existing clinical conditions (*Figure 1B*; Supplementary data online, *Table S1*). Loss of Y chromosome was higher in older patients, in smokers, and in patients with myocardial infarction, while the frequency of LOY was lower in patients with diabetes.

Loss of Y chromosome was dichotomized at a previously published optimized cut-off of 17%.^10^ The prevalence of LOY > 17% was 9.7% (*N* = 165). Baseline characteristics of the two groups according to this cut-off of LOY are shown in Supplementary data online, *Table S2*. Patients with LOY > 17% were significantly older and had a lower body mass index as compared with those with LOY ≤ 17%. Furthermore, the prevalence of smoking, CAD, and myocardial infarction was higher in patients with LOY > 17%. In addition, hsCRP and troponin T levels were significantly higher in patients with LOY > 17%. Medication at baseline did not differ between both groups (see Supplementary data online, *Table S3*).

**Figure 2:**
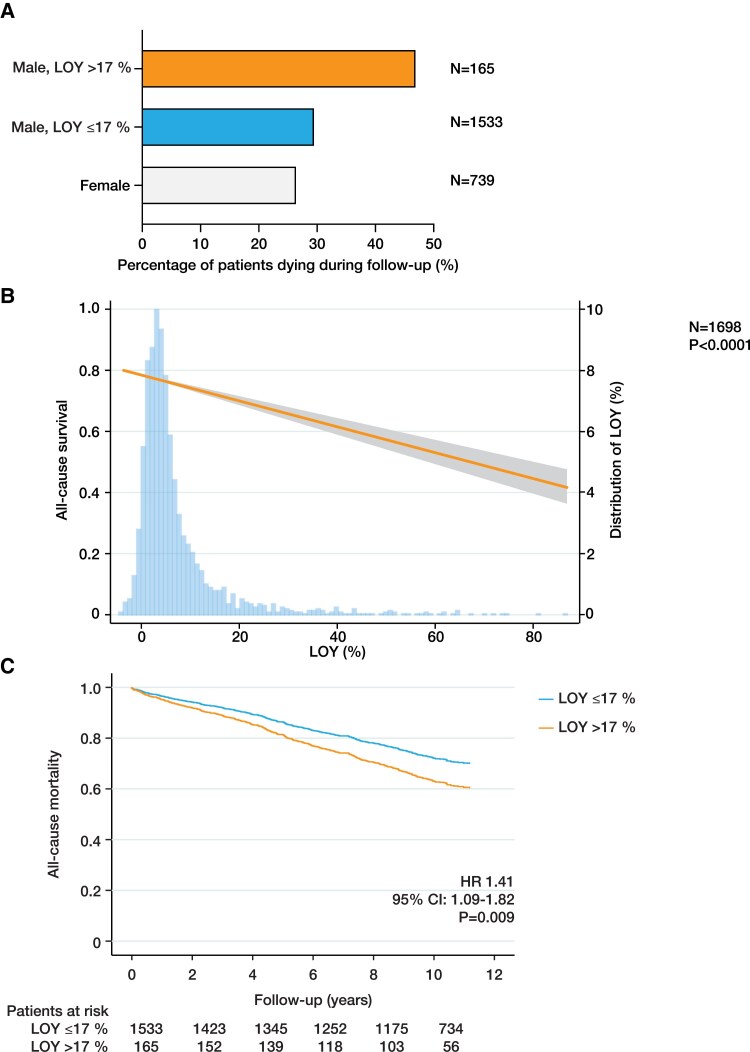
Association between loss of Y chromosome and mortality. (*A*) Unadjusted all-cause mortality rates in females, males with loss of Y chromosome ≤ 17%, and males with loss of Y chromosome > 17% during follow-up. (*B*) Plot of the survival function according to loss of Y chromosome. Analysis is adjusted for age, age^2^, smoking status, body mass index, LDL cholesterol, high-sensitivity C-reactive protein, troponin T, diabetes, hypertension, myocardial infarction, and coronary artery disease. The line indicates mortality with the 95% confidence interval (grey-shaded area). Histogram shows the distribution of loss of Y chromosome (right *Y* axis). (*C*) Survival curve showing the association between loss of Y chromosome and all-cause mortality as determined by Cox regression analyses adjusted for age, age^2^, smoking status, body mass index, LDL cholesterol, high-sensitivity C-reactive protein, troponin T, diabetes, hypertension, myocardial infarction, and coronary artery disease. For the plot, covariates were set to their mean value. HR, hazard ratio

During a median follow-up of 9.9 years (interquartile range 2.2 years), 47.0% of men with LOY > 17% died, while mortality in men with LOY ≤ 17% was 29.6% and comparable with women (*N* = 739; 26.5%; *Figure 2A*). As illustrated in *Figure 2B*, there was a significant association between the extent of LOY as a continuous variable and all-cause mortality. Even after adjustment for potential confounders including age, LOY > 17% was associated with significantly higher all-cause mortality [hazard ratio (HR) 1.41, 95% CI 1.09–1.82, *P* = .009; *Figure 2C*; Supplementary data online, *Table S4*]. Importantly, the association between LOY and all-cause mortality was independent of the selection of a specific LOY cut-off (see Supplementary data online, *Table S5*). In subgroup analyses (see Supplementary data online, *Table S6*), there was a significant interaction between LOY and diabetes (*P* = .035) in the association with all-cause mortality.

### Loss of Y chromosome and cardiovascular mortality

**Figure 3:**
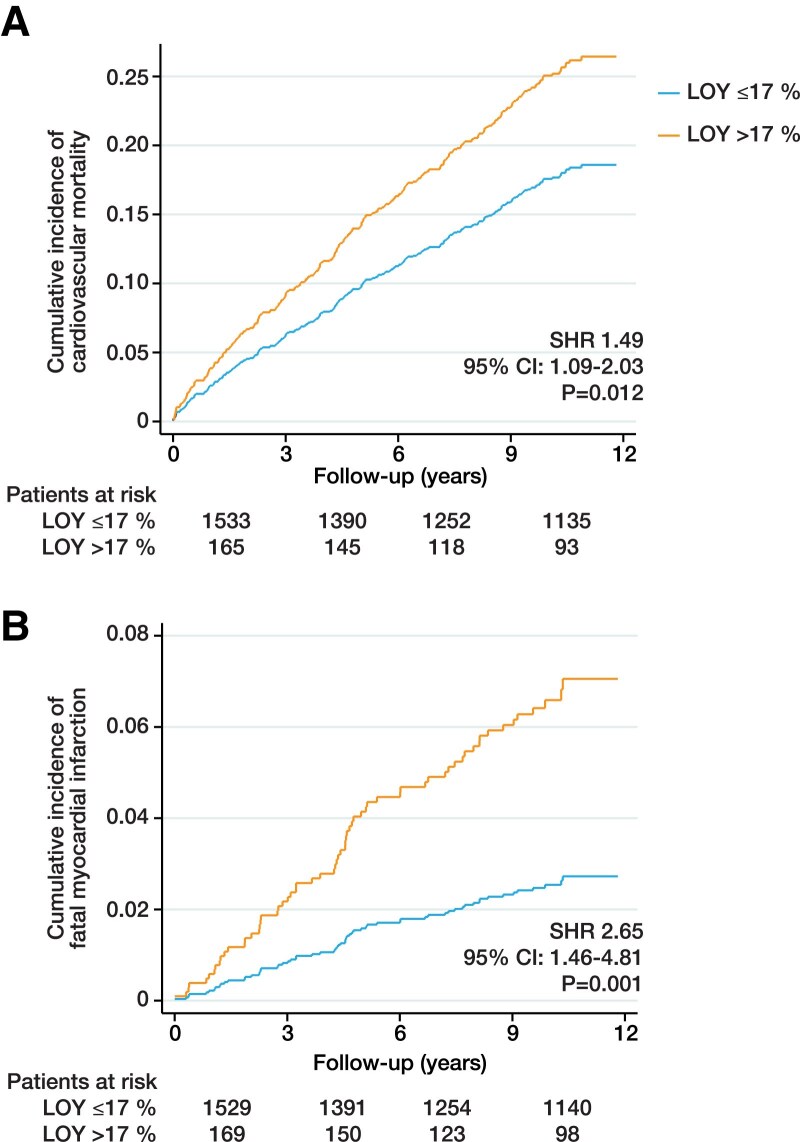
Association between loss of Y chromosome and specific causes of death. Cumulative incidence curves showing the association between loss of Y chromosome and (*A*) cardiovascular mortality and (*B*) death due to fatal myocardial infarction as determined by competing risk regression analyses adjusted for age, age^2^, smoking status, body mass index, LDL cholesterol, high-sensitivity C-reactive protein, troponin T, diabetes, hypertension, myocardial infarction, and coronary artery disease. SHR, subhazard ratio

We next analysed the association between LOY and specific causes of death by using competing risk regression analyses. Importantly, LOY > 17% was significantly associated with higher cardiovascular mortality (SHR 1.49, 95% CI 1.09–2.03, *P* = .012; *Figure 3A*; Supplementary data online, *Table S7*). Consistently, LOY as a continuous variable was also associated with higher cardiovascular mortality (see Supplementary data online, *Table S8*). Interestingly, we found that higher cardiovascular mortality in patients with LOY > 17% was mainly driven by a higher risk for fatal myocardial infarction (SHR 2.65, 95% CI 1.46–4.81, *P* = .001; *Figure 3B*; Supplementary data online, *Tables S9 and S10*). We observed only a trend towards higher cancer-related mortality in patients with LOY > 17% (HR 1.88, 95% CI .98–3.62, *P* = .059; Supplementary data online, *Tables S11 and S12*).

### Loss of Y chromosome associates with a profibrotic and proinflammatory phenotype

**Figure 4:**
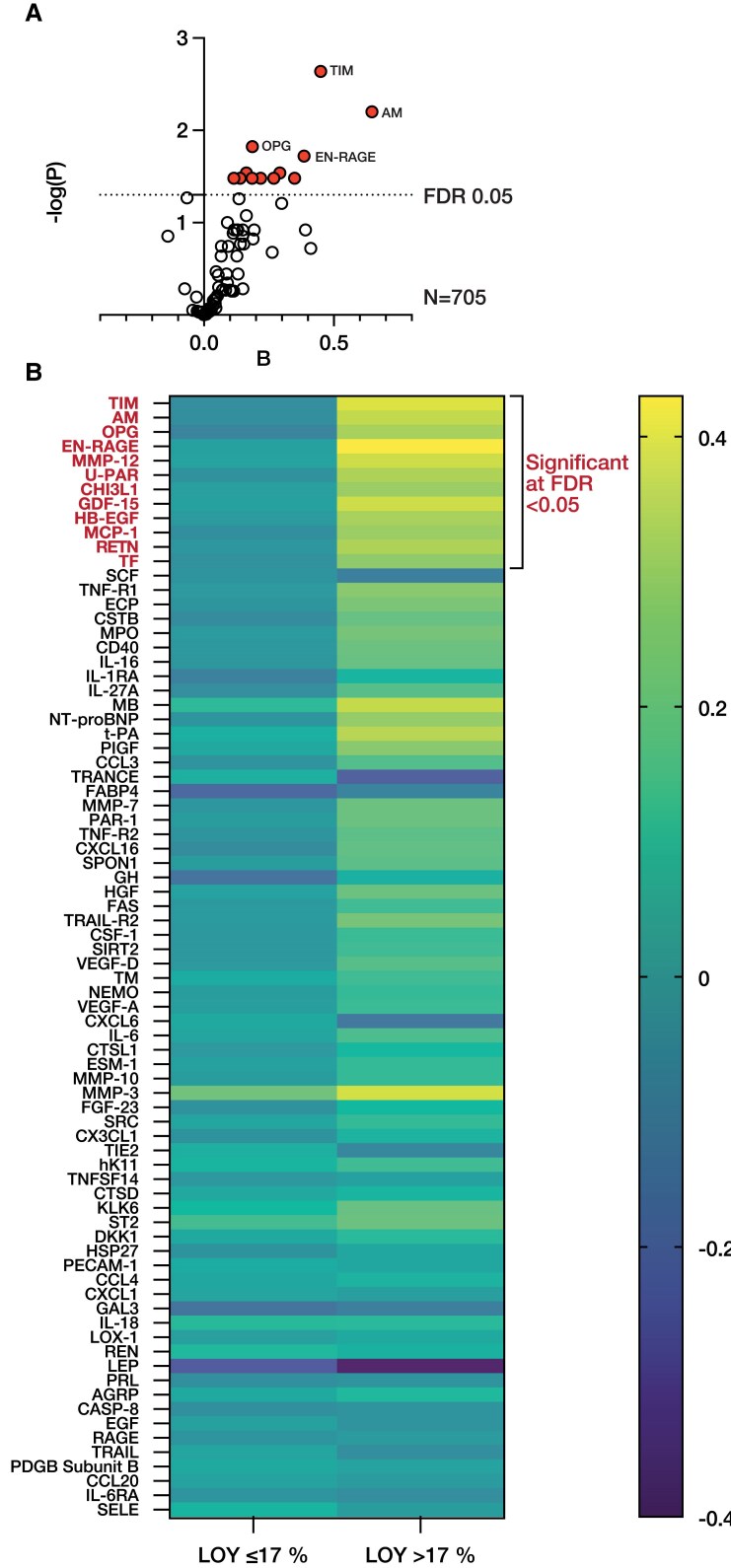
Association between loss of Y chromosome and plasma protein profile. (*A*) Volcano plot showing the effect size (*B*) as well as the significance level of the normalized protein levels of 80 plasma proteins quantified using the OLINK platform in patients with loss of Y chromosome > 17% as compared with those with loss of Y chromosome ≤ 17%. Analyses were adjusted for age, smoking status, body mass index, LDL cholesterol, high-sensitivity C-reactive protein, troponin T, diabetes, hypertension, myocardial infarction, coronary artery disease, and number of NPX values below detection across all proteins. To account for multiple testing, *P*-values were calculated at FDR of .05 (*P*-value*rank/number of comparisons, i.e. *N* = 80). (*B*) Heat map showing protein plasma levels (OLINK normalized protein expression levels transformed as *Z*-scores) according to loss of Y chromosome ≤ 17% and >17%

To gain additional mechanistical insights on how LOY modifies CVD outcomes in patients undergoing coronary angiography, we analysed the differences in cardiovascular protein biomarkers as determined by the OLINK platform according to the frequency of LOY (*Figure 4A*; Supplementary data online, *Table S13*). Loss of Y chromosome > 17% was significantly associated with higher plasma levels of TIM-1, adrenomedullin (AM), osteoprotegerin (OPG), receptor for advanced glycosylation end products (EN-RAGE), matrix metalloproteinase-12 (MMP-12), urokinase plasminogen activator surface receptor (U-PAR), chitinase-3-like protein 1 (CHI3L1), growth/differentiation factor 15 (GDF-15), heparin-binding EGF-like growth factor (HB-EGF), monocyte chemotactic protein-1 (MCP-1), resistin (RETN), and tissue factor (TF, *Figure 4B*).

### Loss of Y chromosome interferes with myocardial fibrosis

We next analysed the interaction between LOY and genetic predisposition to interstitial myocardial fibrosis. For this purpose, we calculated a previously described weighted genetic risk score (wGRS) for myocardial fibrosis (see Supplementary data online, *Table S14*).^15^ The risk score comprises 11 SNPs, which have recently been reported to be associated with higher myocardial T1 intensity as determined by MRI as a measure for diffuse myocardial fibrosis. The wGRS for myocardial fibrosis was neither associated with all-cause nor with cardiovascular mortality in LURIC (see Supplementary data online, *Table S15*).

**Figure 5:**
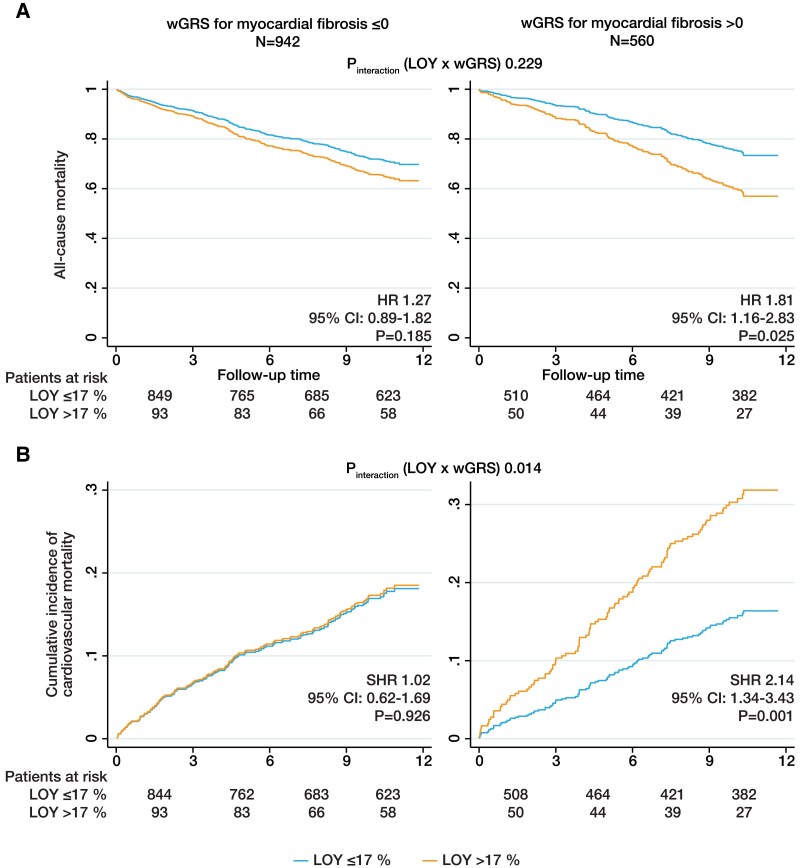
Modulation of the effects of loss of Y chromosome on mortality by myocardial fibrosis. (*A*) Survival curve showing the association between loss of Y chromosome and all-cause mortality and (*B*) cumulative incidence curve showing the association between loss of Y chromosome and cardiovascular mortality according to the weighted genetic risk score for myocardial fibrosis as determined by Cox regression analysis and competing risk regression analysis adjusted for age, age^2^, smoking status, body mass index, LDL cholesterol, high-sensitivity C-reactive protein, troponin T, diabetes, hypertension, myocardial infarction, and coronary artery disease. SHR, subhazard ratio

Interestingly, we found that LOY was only associated with higher all-cause mortality (HR 1.81, 95% CI 1.16–2.83) in patients with myocardial fibrosis wGRS > 0, although the interaction term between LOY and fibrosis wGRS did not reach statistical significance (*P*_interaction_ = .229; *Figure 5A*; Supplementary data online, *Table S16*). Importantly, there was a significant interaction between LOY and the fibrosis wGRS in the prediction of cardiovascular mortality (*P*_interaction_ = .014; *Figure 5B*; Supplementary data online, *Table S17*). Loss of Y chromosome > 17% was strongly associated with higher cardiovascular mortality (SHR 2.14, 95% CI 1.34–3.43) in patients with wGRS > 0, while there was no association in patients with wGRS ≤ 0 (SHR 1.02, 95% CI .62–1.69).

### Loss of Y chromosome associates with altered DNA methylation and profibrotic gene expression

**Figure 6:**
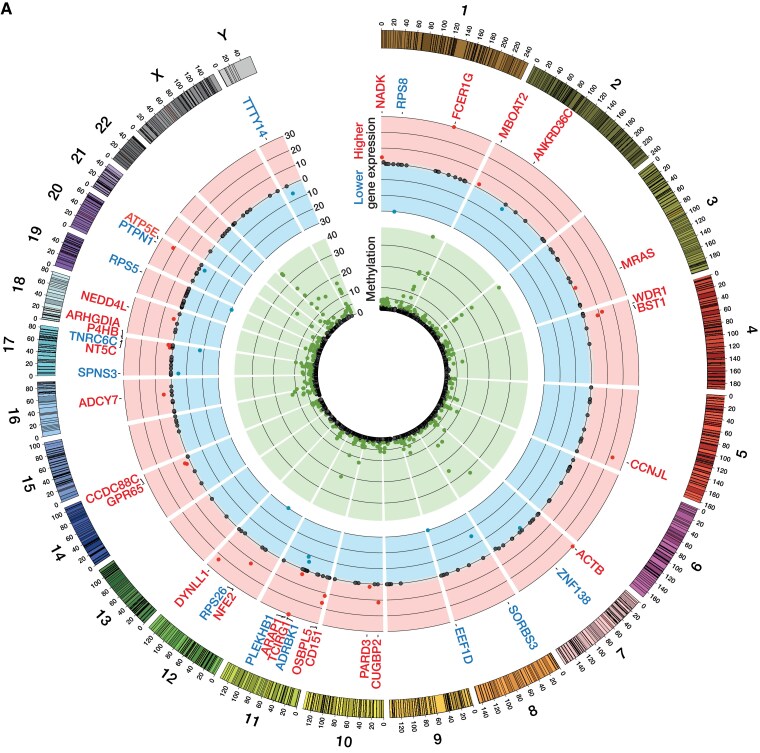
Loss of Y chromosome alters methylation and fibrosis-regulating gene expression. (*A*) Circos plot. Green band corresponds to -log_10_(*P*) for association of loss of Y chromosome with DNA methylation with *Y* axis truncated at 30 by chromosomal position. Red band and red gene labels correspond to -log_10_(*P*) for higher gene expression and blue band and blue gene labels to -log_10_(P) for lower gene expression in loss of Y chromosome peripheral blood mononuclear cells as compared with Y-positive peripheral blood mononuclear cells of the 298 differentially methylated genes as determined by single-cell RNA sequencing

To gain additional insights on how LOY may modulate cardiovascular mortality, we analysed the association between LOY and genome-wide methylation. As shown in *Figure 6A* and Supplementary data online, *Table S18*, LOY was associated with differential methylation of 298 genes. To test the relevance of these findings, we used scRNAseq to determine on whether differential methylation of these genes associates with altered gene expression in Y-positive as compared with LOY PBMCs. Of the 298 differentially methylated genes, mRNA expression of 37 genes differed significantly in Y-positive vs. LOY PBMCs (*Figure 6A*; Supplementary data online, *Table S19*). Thereof, 12 genes were significantly down-regulated in LOY PBMCs, whereas 25 genes were significantly up-regulated. Interestingly, several of these genes including *ARAP1*, *BST1*, *CD151*, *DYNLL1*, *FCER1G*, *NADK1*, *RPS5*, and *WDR1* have been recently shown to be involved in the development of organ fibrosis.^20–28^

### Loss of Y chromosome target gene *RPS5* promotes a paracrine profibrotic phenotype in human cardiac fibroblasts

**Figure 7:**
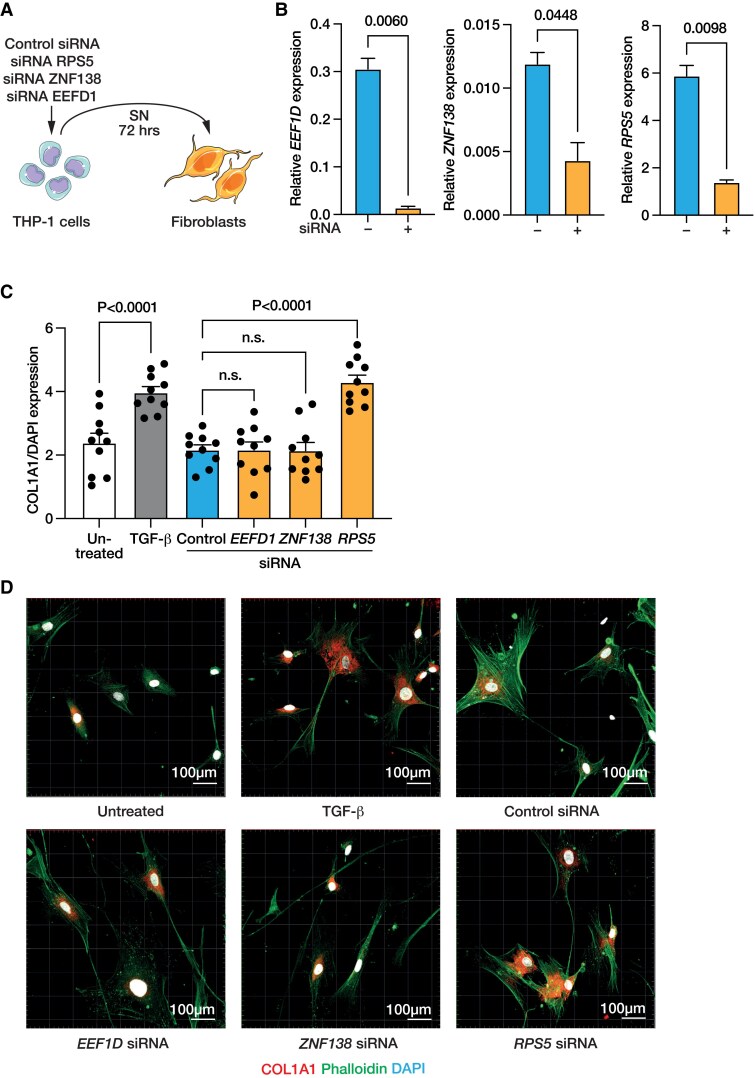
Knockdown of the loss of Y chromosome target gene *RPS5* induces a profibrotic response. (*A*) Experimental outline. (*B*) Gene expression in THP1-derived macrophages after small interfering RNA-mediated knockdown of the loss of Y chromosome target genes *EEF1D*, *ZNF138*, and *RPS5* to determine knockdown efficiency. (*C*) Quantification of the COL1A1-positive area normalized to DAPI-positive area in cardiac fibroblasts incubated for 72 h with supernatant of THP1-derived macrophages after knockdown of *EEF1D*, *ZNF138*, or *RPS5*, respectively, or transforming growth factor-β (50 ng/mL) as positive control. (*D*) Representative confocal images of cardiac fibroblasts. SN, supernatant

To examine the relevance of these findings, we selected three genes that were differentially methylated in patients with LOY > 17% and down-regulated in the scRNAseq data set, namely, *EEF1D*, *ZNF138*, and *RPS5*. We performed siRNA-mediated knockdown of these genes in THP1-derived macrophages, collected the supernatant, and incubated HCFs with this supernatant for 72 h (*Figure 7A and B*). Interestingly, as shown in *Figure 7C and D*, we found that treatment of fibroblasts with THP-1 supernatant after *RPS5*-knockdown promoted accumulation of collagen 1A1, which indicates that down-regulation of *RPS5* to mimic the effects observed under LOY *in vitro* indeed induces a paracrine profibrotic response in HCFs.

## Discussion

The results of the present study identify LOY as a novel independent risk factor for adverse outcomes in patients undergoing coronary angiography. Loss of Y chromosome was associated with a higher risk for mortality, which was mainly driven by a higher risk for cardiovascular mortality and in particular fatal myocardial infarction. Furthermore, we found that LOY was associated with a profibrotic-proinflammatory protein signature in the plasma. Mechanistically, LOY was associated with differential methylation and gene expression of several fibrosis-regulating genes. Finally, germline predisposition to diminished myocardial fibrosis abrogated the LOY-associated increased mortality. Moreover, lower *RPS5* expression in LOY leukocytes induced collagen-1 accumulation in cardiac fibroblasts (*Structured Graphical Abstract*).

Loss of Y chromosome has recently been identified as a risk factor for age-associated diseases such as Alzheimer’s disease, CVD, and cancer in patients without prevalent CVD on the population level.^6,29,30^ In LURIC, a well-characterized cohort of patients undergoing coronary angiography with a CAD prevalence of 82.9%, LOY was associated with higher all-cause mortality, cardiovascular mortality, and death due to cancer during long-term follow-up. Importantly, the association persisted even after adjustment for potential confounders including age. For the present analyses, we used a previously described cut-off of 17% LOY. Nevertheless, our analyses were independent of the selection of a specific cut-off of LOY, since LOY as a continuous variable was also consistently associated with all mortality endpoints in LURIC. Furthermore, we found a significant interaction between LOY and markers of inflammation as well as smoking indicating that the detrimental cardiovascular effects of LOY can be enhanced by these conditions and that currently emerging anti-inflammatory therapies^5^ or cessation of smoking might provide a particular benefit in patients with LOY. This is further supported by the observation that smoking itself was linked to higher frequency of LOY in LURIC, which is in line with a previous report.^8^

Using the OLINK multiplex CVD protein array, we detected higher plasma levels of TIM, AM, OPG, EN-RAGE, MMP-12, U-PAR, CHI3L1, GDF-15, HB-EGF, MCP-1, RETN, and TF in patients with LOY ≥ 17%. Of note, OPG and MMP-12 are both involved in the development of endothelial dysfunction, atherosclerotic lesions, and myocardial fibrosis.^31–33^ Interestingly, MMP-12 might be of particular importance in males, since oestrogens may down-regulate MMP-12 expression in macrophages, thereby reducing arterial stiffness and atherosclerotic lesion size.^34^ Although Mendelian randomization analyses found that MMP-12 was associated with a lower risk for ischaemic stroke,^35^ proteomic analyses in UK Biobank revealed a significant association between MMP-12 plasma levels and a higher risk for incident CAD.^36^ Moreover, U-PAR,^37^ CHI3L1,^38^ GDF-15,^39^ HB-EGF,^40^ MCP-1,^41^ and Retn^42^ all have been documented to be involved in the development of organ fibrosis. These findings indicate the presence of a profibrotic and proinflammatory signature in the blood of patients with LOY. Future studies have to elucidate whether this represents an association with or a consequence of LOY.

It has been previously experimentally documented that haematopoietic LOY represents a direct driver of cardiac fibrosis.^9^ In line with these findings, we previously detected a profibrotic gene signature in monocytes from patients with LOY.^10^ Moreover, our very recent study disclosed that LOY not only associates with increased serum markers of organ fibrosis but also with increased mortality in patients with chronic kidney disease.^11^ Indeed, myocardial fibrosis represents a common denominator in various forms of CVD including ischaemic and non-ischaemic heart disease.^43,44^ Recently, it has been documented that increased native myocardial T1 time, a measure for diffuse myocardial interstitial fibrosis, as determined by cardiac magnetic resonance imaging was associated with major adverse cardiovascular events at the population level.^15^ In this study, SNPs in 11 gene loci were identified to be significantly genome-wide associated with increased myocardial T1 time. Based on these findings, we built a wGRS for myocardial fibrosis in the present study in order to quantify the germline predisposition to cardiac fibrosis. Notably, in LURIC, there was no association between this wGRS and cardiovascular mortality. However, we found a significant interaction between the myocardial fibrosis wGRS and LOY. A lower inherited genetic predisposition for myocardial fibrosis completely abolished the association between LOY and cardiovascular mortality. These findings indicate that the LOY-associated adverse outcomes in LURIC might by related to an enhanced genetic susceptibility to cardiac fibrosis.

Our combined analysis of DNA methylation and gene expression identified several candidate genes including *ARAP1*, *BST1*, *CD151*, *DYNLL1*, *NADK1*, *WDR1*, and *RPS5*, of which several are known to be involved in fibrosis regulation.^20–28^ Experimental analyses of three down-regulated genes documented a paracrine activation of fibroblasts by silencing of *RPS5* in macrophages, whereas silencing of *EEFD1* and *ZNF138* did not show an effect in this *in vitro* screen. Little is known regarding the role of the ribosomal protein RPS5 in macrophages, but it has been proposed to play a role in pathogen-mediated activation of macrophages.^45^ Moreover, knockdown of *RPS5* has been shown to aggravate liver fibrosis^20^ and, vice versa, up-regulation of *RPS5* ameliorated cardiac fibrosis in mice.^21^ Indeed, our additional *in vitro* experiments revealed that treatment of HCFs with supernatant of macrophages after knockdown of *RPS5* induced significant collagen deposition. Thus, the results of the present study further support a significant contribution of profibrotic mechanisms to LOY-mediated cardiovascular injury.

Our study is not without limitations. The LURIC study mainly included patients of Caucasian ancestry and is enriched for patients with CAD. Therefore, the results cannot be extrapolated to the general population and to other ethnicities. Furthermore, due to the study design, the LURIC study does not provide non-fatal cardiovascular endpoints. Additionally, although our analyses establish robust associations between LOY and cardiovascular mortality, it cannot infer a causal relationship. Moreover, it should be the subject of future studies to assess whether patients with LOY will particularly benefit from therapies modulating profibrotic mechanisms such as sodium-glucose co-transporter 2 inhibitors,^46^ selective non-steroidal mineralocorticoid receptor antagonists,^47^ or novel anti-fibrotic therapeutic approaches including specific inhibitors of TGF-ß.^48^ Furthermore, the findings on the interaction between LOY and the wGRS for myocardial fibrosis should be independently replicated.

Overall, the present study identified the age-associated LOY in circulating blood cells as an important independent risk factor for adverse outcomes in men with prevalent CAD. These findings are of particular relevance, given the continuously growing elderly population. Quantification of LOY may aid in the identification of patients, who will particularly benefit from intensified secondary preventive therapeutic strategies as a sex-specific personalized medicine approach.

## Supplementary Material

ehaf035_Supplementary_Data

## Declarations

### Data Availability

Data are available from the corresponding author upon request.

### Pre-registered Clinical Trial Number

None supplied.
