# Supplementary material for: Mosaic loss of Y chromosome and mortality after coronary angiography
Source: Eur Heart J. 2025 Feb 12;46(17):1603–16. doi: 10.1093/eurheartj/ehaf035 (PMC12046225; doi:10.1093/eurheartj/ehaf035)
Supplement: ehaf035_Supplementary_Data [file ehaf035_supplementary_data.docx]

**Supplemental figures**

**Supplemental Figure 1**

**(A)** Workflow showing patients of LURIC at each step of analysis. **(B)** Directed acyclic graph.


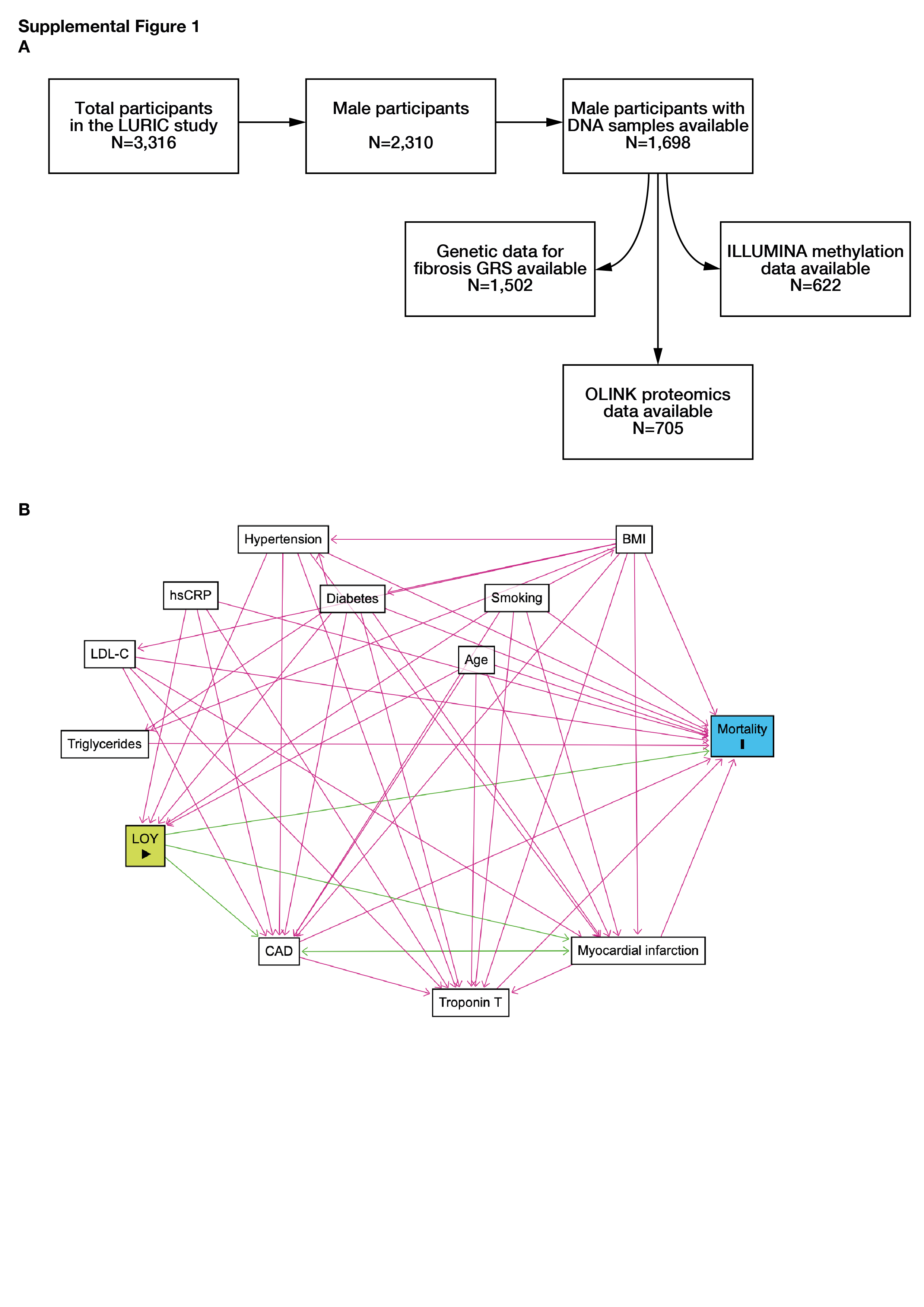


**Supplemental tables**

**Supplemental Table 1**

**Association of baseline characteristics with LOY**

| **Parameter** | **Category** | **N** | **LOY % (95 % CI)** | **P** |
| --- | --- | --- | --- | --- |
| **Age (years)** | ≤63.5 | 912 | 5.1 (4.0-6.3) | <0.001 |
|  | >63.5 | 786 | 9.7 (8.5-10.9) |  |
| **Testosterone (nmol/L)** | ≤8.7 | 149 | 7.8 (5.8-9.7) | 0.470 |
|  | 8.8-17.0 | 752 | 7.0 (5.9-8.0) |  |
|  | >17.0 | 797 | 7.5 (6.5-8.6) |  |
| **Triglycerides (mg/dL)** | ≤121 | 545 | 7.7 (6.5-9.0) | 0.339 |
|  | 122-179 | 570 | 7.7 (6.4-8.9) |  |
|  | >179 | 583 | 6.9 (5.6-8.2) |  |
| **LDL-C (mg/dL)** | ≤101 | 598 | 7.8 (6.6-9.0) | 0.289 |
|  | 102-130 | 593 | 6.9 (5.7-8.1) |  |
|  | >130 | 507 | 7.6 (6.3-8.9) |  |
| **Glycated hemoglobin (%)** | ≤5.70 | 589 | 7.0 (5.7-8.3) | 0.408 |
|  | 5.71-6.30 | 559 | 7.7 (6.5-9.0) |  |
|  | >6.30 | 550 | 7.6 (6.4-8.8) |  |
| **Friesinger Score** | ≤3 | 523 | 7.6 (6.4-8.8) | 0.951 |
|  | 4-7 | 562 | 7.4 (5.9-8.8) |  |
|  | >7 | 613 | 7.3 (5.9-8.7) |  |
| **Troponin T (pg/mL)** | ≤7.0 | 530 | 7.5 (6.1-8.8) | 0.954 |
|  | 7.1-18.0 | 554 | 7.3 (6.1-8.6) |  |
|  | >18.0 | 614 | 7.5 (6.3-8.7) |  |
| **Diabetes** | No | 1164 | 8.1 (7.0-9.1) | 0.047 |
|  | Yes | 534 | 6.8 (5.5-8.1) |  |
| **Hypertension** | No | 1218 | 7.2 (5.9-8.5) | 0.504 |
|  | Yes | 480 | 7.6 (6.6-8.7) |  |
| **Coronary artery disease** | No | 309 | 7.2 (5.5-8.9) | 0.578 |
|  | Yes | 1389 | 7.7 (6.8-8.6) |  |
| **Smoking** | No | 390 | 6.5 (5.5-7.5) | 0.017 |
|  | Yes | 1308 | 7.9 (7.4-8.4) |  |
| **Myocardial infarction** | No | 912 | 6.7 (5.6-7.8) | 0.020 |
|  | Yes | 786 | 8.1 (6.9-9.4) |  |
| **Lipid-lowering therapy** | No | 845 | 7.8 (6.6-8.9) | 0.245 |
|  | Yes | 853 | 7.1 (5.9-8.3) |  |
| **hsCRP (mg/L)** | ≤1.8 | 577 | 6.7 (5.4-7.9) | 0.057 |
|  | 1.9-6.2 | 548 | 7.3 (6.1-8.6) |  |
|  | >6.2 | 573 | 8.3 (7.0-9.6) |  |

Adjusted for age, testosterone, triglycerides, LDL-C, glycated hemoglobin, Friesinger score, troponin T, diabetes, hypertension, coronary artery disease, smoking, myocardial infarction, lipid-lowering therapy, smoking, hsCRP.

**Supplemental table 2**

**Baseline characteristics**

|  | **Female participants** | **Male participants** | | **P*** |
| --- | --- | --- | --- | --- |
|  |  | **LOY** | |  |
|  | **N=739** | **≤17 %**  **N=1533** | **>17 %**  **N=165** |  |
| **Age (years)** | 65.2 (58.6-65.2) | 61.8 (54.1-69.0) | 69.9 (63.3-74.9) | <0.001 |
| **BMI (kg/m^2^)** | 26.9 (24.1-30.1) | 27.1 (25.0-29.6) | 26.3 (24.5-28.6) | 0.010 |
| **Smoking (%)** | 35.8 | 76.6 | 81.9 | 0.001 |
| **Hypertension (%)** | 76.0 | 71.1 | 78.3 | 0.056 |
| **Diabetes mellitus (%)** | 32.6 | 39.2 | 45.2 | 0.132 |
| **Testosterone (nmol/L)** | 2.8 (1.7-3.5) | 16.7 (12.5-21.2) | 16.5 (13.2-21.5) | 0.970 |
| **hsCRP (mg/L)** | 3.6 (1.5-8.7) | 3.4 (1.2-8.6) | 3.9 (1.6-10.3) | 0.005 |
| **Triglycerides (mg/dL)** | 140 (105-201) | 151 (112-202) | 133 (104-188) | 0.117 |
| **LDL-C (mg/dL)** | 120 (99-145) | 112 (92-134) | 114 (89-140) | 0.689 |
| **Glycated hemoglobin (%)** | 6.10 (5.60-6.70) | 6.00 (5.60-6.60) | 6.10 (5.70-6.63) | 0.234 |
| **Friesinger score** | 3.0 (0.0-7.0) | 6.0 (3.0-9.0) | 7.0 (4.0-9.0) | 0.025 |
| **Coronary artery disease (%)** | 64.7 | 82.0 | 91.5 | 0.001 |
| **Myocardial infarction (%)** | 28.5 | 45.0 | 57.8 | 0.002 |
| **Troponin T (pg/mL)** | 8.0 (3.0-18.8) | 12.0 (6.0-28.0) | 14.0 (7.8-33.1) | 0.042 |
| **Lipid-lowering therapy (%)** | 43.7 | 50.4 | 48.5 | 0.682 |

Continuous variables are shown as median (25 %-75 % quantiles)

* for the comparision between LOY ≤17 % vs. LOY >17 %

**Supplemental table 3**

**Medication at baseline (%) according to LOY**

| **Medication** | **LOY ≤17 %** | **LOY >17 %** | **P** |
| --- | --- | --- | --- |
| Aspirin or other antiplatelet agent (%) | 73.5 | 71.7 | 0.644 |
| ACE inhibitor (%) | 55.4 | 53.0 | 0.566 |
| Beta blocker (%) | 64.0 | 65.7 | 0.733 |
| Angiotensin-2 receptor blocker (%) | 3.8 | 3.6 | 0.999 |
| Calcium antagonist (%) | 14.9 | 16.9 | 0.493 |
| Oral antidiabetics (%) | 8.7 | 6.6 | 0.463 |
| Insulin treatment (%) | 4.4 | 4.2 | 0.999 |
| Lipid-lowering therapy (%) | 50.4 | 48.5 | 0.682 |

**Supplemental table 4**

**Association between LOY and all-cause mortality**

| **Model** | **LOY** | **HR (95% CI)** | **P** |
| --- | --- | --- | --- |
| Model 1 | ≤17 % | Reference | |
|  | >17 % | 1.36 (1.06-1.74) | 0.016 |
| Model 2 | ≤17 % | Reference | |
|  | >17 % | 1.41 (1.09-1.82) | 0.009 |

Model 1: adjusted for age, age^2^, diabetes, hypertension, smoking, and hsCRP

Model 2: adjusted for age, age^2^, smoking, body mass index, LDL-C, hsCRP, troponin T, diabetes, hypertension, myocardial infarction, coronary artery disease

**Supplemental table 5**

**Association between LOY as continuous variable (transformed as z-score) and all-cause mortality**

| **Model** | **HR (95% CI) per unit** | **P** |
| --- | --- | --- |
| Model 1 | 1.11 (1.03-1.19) | 0.0058 |
| Model 2 | 1.11 (1.03-1.19) | 0.0065 |

Model 1: adjusted for age, age^2^, diabetes, hypertension, smoking, and hsCRP

Model 2: adjusted for age, age^2^, smoking, body mass index, LDL-C, hsCRP, troponin T, diabetes, hypertension, myocardial infarction, coronary artery disease

**Supplemental table 6**

**Association between LOY and all-cause mortality according to baseline characteristics**

| **LOY** |  | |  | |  |
| --- | --- | --- | --- | --- | --- |
|  | **HR (95% CI)** | **P** | **HR (95% CI)** | **P** | **P_interaction_** |
|  | **No Diabetes** | | **Diabetes** | |  |
| >17 % | 1.58 (1.13-2.20) | 0.0073 | 1.09 (0.72-1.66) | 0.684 | 0.035 |
|  | **No CAD** | | **CAD** | |  |
| >17 % | 1.21 (0.33-4.44) | 0.775 | 1.44 (1.11-1.87) | 0.0061 | 0.067 |
|  | **No Smoking** | | **Smoking** | |  |
| >17 % | 1.06 (0.52-2.16) | 0.877 | 1.53 (1.16-2.02) | 0.0026 | 0.076 |
|  | **No myocardial infarction** | | **Myocardial infarction** | |  |
| >17 % | 1.23 (0.80-1.88) | 0.340 | 1.54 (1.11-2.13) | 0.0092 | 0.495 |
|  | **No hypertension** | | **Hypertension** | |  |
| >17 % | 1.25 (0.66-2.38) | 0.501 | 1.50 (1.13-1.98) | 0.0050 | 0.810 |
|  | **hsCRP <3.4 mg/L** | | **hsCRP ≥3.4 mg/L** | |  |
| >17 % | 0.92 (0.58-1.45) | 0.722 | 1.77 (1.30-2.43) | 0.0004 | 0.060 |
|  | **LDL-C ≤114 mg/dL** | | **LDL-C >115 mg/dL** | |  |
| >17 % | 1.36 (0.94-1.96) | 0.102 | 1.55 (1.07-2.22) | 0.019 | 0.823 |
|  | **Age ≤63.5 years** | | **Age >63.5 years** | |  |
| >17 % | 1.24 (0.61-2.54) | 0.558 | 1.36 (1.04-1.79) | 0.026 | 0.811 |
|  | **BMI ≤27.0 kg/m2** | | **BMI >27.0 kg/m^2^** | |  |
| >17 % | 1.44 (1.03-2.02) | 0.035 | 1.49 (0.99-2.23) | 0.054 | 0.560 |

Given are the hazard ratios for the comparison between LOY ≤17 % (reference) vs. LOY>17 %

Adjusted for age, age^2^. smoking status, body mass index, LDL-C, hsCRP, troponin T, diabetes, hypertension, myocardial infarction, coronary artery disease.

**Supplemental table 7**

**Association between LOY and cardiovascular mortality**

| **Model** | **LOY** | **SHR (95% CI)** | **P** |
| --- | --- | --- | --- |
| Model 1 | ≤17 % | Reference | |
|  | >17 % | 1.50 (1.10-2.05) | 0.011 |
| Model 2 | ≤17 % | Reference | |
|  | >17 % | 1.49 (1.09-2.03) | 0.012 |

Model 1: adjusted for age, age^2^, diabetes, hypertension, smoking, and hsCRP

Model 2: adjusted for age, age^2^, smoking, body mass index, LDL-C, hsCRP, troponin T, diabetes, hypertension, myocardial infarction, coronary artery disease

SHR. subhazard ratio

**Supplemental table 8**

**Association between LOY as continuous variable (transformed as z-score) and cardiovascular mortality**

| **Model** | **SHR (95% CI)** | **P** |
| --- | --- | --- |
| Model 1 | 1.14 (1.05-1.24) | 0.002 |
| Model 2 | 1.14 (1.05-1.25) | 0.002 |

Model 1: adjusted for age, age^2^, diabetes, hypertension, smoking, and hsCRP

Model 2: adjusted for age, age^2^, smoking, body mass index, LDL-C, hsCRP, troponin T, diabetes, hypertension, myocardial infarction, coronary artery disease

SHR. subhazard ratio

**Supplemental table 9**

**Association between LOY and fatal myocardial infarction**

| **Model** | **LOY** | **SHR (95% CI)** | **P** |
| --- | --- | --- | --- |
| Model 1 | ≤17 % | Reference | |
|  | >17 % | 2.70 (1.51-4.85) | 0.001 |
| Model 2 | ≤17 % | Reference | |
|  | >17 % | 2.65 (1.46-4.81) | 0.001 |

Model 1: adjusted for age, age^2^, diabetes, hypertension, smoking, and hsCRP

Model 2: adjusted for age, age^2^, smoking, body mass index, LDL-C, hsCRP, troponin T, diabetes, hypertension, myocardial infarction, coronary artery disease

SHR. subhazard ratio

**Supplemental table 10**

**Association between LOY as continuous variable (transformed as z-score) and fatal myocardial infarction**

| **Model** | **SHR (95% CI)** | **P** |
| --- | --- | --- |
| Model 1 | 1.32 (1.15-1.53) | <0.001 |
| Model 2 | 1.32 (1.15-1.52) | <0.001 |

Model 1: adjusted for age, age^2^, diabetes, hypertension, smoking, and hsCRP

Model 2: adjusted for age, age^2^, smoking, body mass index, LDL-C, hsCRP, troponin T, diabetes, hypertension, myocardial infarction, coronary artery disease

SHR. subhazard ratio

**Supplemental table 11**

**Association between LOY and cancer mortality**

| **Model** | **LOY** | **SHR (95% CI)** | **P** |
| --- | --- | --- | --- |
| Model 1 | ≤17 % | Reference | |
|  | >17 % | 1.62 (0.86-3.08) | 0.138 |
| Model 2 | ≤17 % | Reference | |
|  | >17 % | 1.88 (0.98-3.62) | 0.059 |

Model 1: adjusted for age, age^2^, diabetes, hypertension, smoking, and hsCRP

Model 2: adjusted for age, age^2^, smoking, body mass index, LDL-C, hsCRP, troponin T, diabetes, hypertension, myocardial infarction, coronary artery disease

SHR. subhazard ratio

**Supplemental table 12**

**Association between LOY as continuous variable (transformed a z-score) and cancer mortality**

| **Model** | **SHR (95% CI)** | **P** |
| --- | --- | --- |
| Model 1 | 1.09 (0.93-1.28) | 0.286 |
| Model 2 | 1.10 (0.93-1.31) | 0.257 |

Model 1: adjusted for age, age^2^, diabetes, hypertension, smoking, and hsCRP

Model 2: adjusted for age, age^2^, smoking, body mass index, LDL-C, hsCRP, troponin T, diabetes, hypertension, myocardial infarction, coronary artery disease

SHR. subhazard ratio

**Supplemental table 13**

**Least square means of multivariate adjusted normalized plasma protein expression (NPX) levels according to LOY (LOY ≤17 % as reference)**

| **Protein** | **N** | **B of NPX** | **SE** | **P** | **Critical value** | **Benjamini-Hochberg Adjusted P value** | **Signifi-cant at FDR 0.05** |
| --- | --- | --- | --- | --- | --- | --- | --- |
| TIM | 705 | 0.449 | 0.1074 | 0.000029 | 0.00063 | 0.0023 | Yes |
| AM | 705 | 0.647 | 0.092 | 0.00016 | 0.0013 | 0.0063 | Yes |
| OPG | 705 | 0.186 | 0.0541 | 0.00058 | 0.0019 | 0.015 | Yes |
| EN-RAGE | 705 | 0.385 | 0.1167 | 0.00098 | 0.0025 | 0.019 | Yes |
| MMP-12 | 705 | 0.292 | 0.0953 | 0.0022 | 0.0038 | 0.029 | Yes |
| U-PAR | 705 | 0.163 | 0.0515 | 0.002 | 0.0032 | 0.029 | Yes |
| CHI3L1 | 704 | 0.349 | 0.1241 | 0.005 | 0.0063 | 0.033 | Yes |
| GDF15 | 705 | 0.269 | 0.0891 | 0.003 | 0.0044 | 0.033 | Yes |
| HB-EGF | 705 | 0.218 | 0.078 | 0.005 | 0.0076 | 0.033 | Yes |
| MCP-1 | 705 | 0.139 | 0.0486 | 0.004 | 0.0057 | 0.033 | Yes |
| RETN | 705 | 0.185 | 0.0639 | 0.004 | 0.0051 | 0.033 | Yes |
| TF | 705 | 0.115 | 0.0411 | 0.005 | 0.0070 | 0.033 | Yes |
| SCF | 705 | -0.065 | 0.0666 | 0.33 | 0.030 | 0.054 | No |
| TNF-R1 | 705 | 0.135 | 0.515 | 0.009 | 0.0082 | 0.055 | No |
| ECP | 702 | 0.299 | 0.1181 | 0.011 | 0.0089 | 0.062 | No |
| CSTB | 699 | 0.163 | 0.0675 | 0.016 | 0.0095 | 0.084 | No |
| MPO | 705 | 0.09 | 0.392 | 0.021 | 0.010 | 0.1 | No |
| CD40 | 705 | 0.129 | 0.0623 | 0.039 | 0.015 | 0.12 | No |
| IL-16 | 661 | 0.127 | 0.0579 | 0.028 | 0.011 | 0.12 | No |
| IL-1RA | 701 | 0.193 | 0.0891 | 0.031 | 0.012 | 0.12 | No |
| IL27-A | 704 | 0.116 | 0.0534 | 0.03 | 0.012 | 0.12 | No |
| MB | 705 | 0.149 | 0.069 | 0.031 | 0.013 | 0.12 | No |
| NT-proBNP | 555 | 0.391 | 0.1912 | 0.041 | 0.016 | 0.12 | No |
| t-PA | 705 | 0.13 | 0.063 | 0.039 | 0.016 | 0.12 | No |
| PIGF | 705 | 0.112 | 0.0555 | 0.044 | 0.017 | 0.13 | No |
| CCL3 | 705 | 0.149 | 0.751 | 0.048 | 0.018 | 0.14 | No |
| TRANCE | 674 | -0.139 | 0.0712 | 0.052 | 0.018 | 0.14 | No |
| FABP4 | 700 | 0.189 | 0.0994 | 0.057 | 0.019 | 0.15 | No |
| MMP-7 | 705 | 0.153 | 0.0721 | 0.034 | 0.015 | 0.17 | No |
| PAR-1 | 705 | 0.14 | 0.063 | 0.026 | 0.011 | 0.17 | No |
| TNF-R2 | 705 | 0.14 | 0.0655 | 0.033 | 0.014 | 0.17 | No |
| CXCL16 | 703 | 0.067 | 0.0375 | 0.072 | 0.020 | 0.18 | No |
| SPON1 | 684 | 0.094 | 0.0522 | 0.072 | 0.021 | 0.18 | No |
| GH | 692 | 0.411 | 0.2335 | 0.078 | 0.021 | 0.19 | No |
| HGF | 705 | 0.263 | 0.1544 | 0.089 | 0.022 | 0.21 | No |
| FAS | 705 | 0.066 | 0.0407 | 0.106 | 0.023 | 0.23 | No |
| TRAIL-R2 | 705 | 0.127 | 0.0784 | 0.106 | 0.022 | 0.23 | No |
| CSF-1 | 705 | 0.047 | 0.0336 | 0.159 | 0.023 | 0.34 | No |
| SIRT2 | 549 | 0.131 | 0.0968 | 0.177 | 0.025 | 0.36 | No |
| VEGF-D | 705 | 0.087 | 0.0647 | 0.177 | 0.024 | 0.36 | No |
| TM | 705 | 0.055 | 0.0416 | 0.187 | 0.025 | 0.37 | No |
| NEMO | 694 | 0.09 | 0.0748 | 0.231 | 0.026 | 0.45 | No |
| VEGF-A | 705 | 0.056 | 0.0503 | 0.267 | 0.027 | 0.5 | No |
| CXCL6 | 705 | -0.074 | 0.0704 | 0.29 | 0.028 | 0.52 | No |
| IL-6 | 652 | 0.15 | 0.1394 | 0.282 | 0.027 | 0.52 | No |
| CTSL1 | 705 | 0.073 | 0.07 | 0.3 | 0.028 | 0.53 | No |
| ESM-1 | 704 | 0.1 | 0.1027 | 0.332 | 0.031 | 0.54 | No |
| MMP-10 | 705 | 0.071 | 0.0734 | 0.33 | 0.030 | 0.54 | No |
| MMP-3 | 704 | 0.083 | 0.0823 | 0.313 | 0.029 | 0.54 | No |
| FGF-23 | 705 | 0.106 | 0.1153 | 0.356 | 0.032 | 0.55 | No |
| SRC | 705 | 0.114 | 0.1222 | 0.352 | 0.032 | 0.55 | No |
| CX3CL1 | 705 | 0.052 | 0.0632 | 0.407 | 0.033 | 0.62 | No |
| TIE2 | 625 | -0.029 | 0.0361 | 0.428 | 0.034 | 0.64 | No |
| hK11 | 705 | 0.046 | 0.0609 | 0.454 | 0.034 | 0.66 | No |
| TNFSF14 | 653 | 0.042 | 0.0573 | 0.465 | 0.035 | 0.67 | No |
| CTSD | 705 | 0.036 | 0.054 | 0.505 | 0.035 | 0.7 | No |
| KLK6 | 705 | 0.038 | 0.0567 | 0.507 | 0.036 | 0.7 | No |
| ST2 | 705 | 0.043 | 0.0711 | 0.549 | 0.037 | 0.75 | No |
| Dkk-1 | 705 | 0.033 | 0.062 | 0.594 | 0.037 | 0.8 | No |
| HSP 27 | 529 | 0.046 | 0.0975 | 0.639 | 0.038 | 0.84 | No |
| PECAM-1 | 705 | 0.017 | 0.0379 | 0.662 | 0.039 | 0.86 | No |
| CCL4 | 705 | 0.025 | 0.0784 | 0.751 | 0.042 | 0.89 | No |
| CXCL1 | 705 | 0.028 | 0.0838 | 0.742 | 0.041 | 0.89 | No |
| Gal-3 | 704 | 0.015 | 0.0441 | 0.739 | 0.041 | 0.89 | No |
| IL-18 | 705 | 0.023 | 0.0656 | 0.729 | 0.040 | 0.89 | No |
| LOX-1 | 705 | 0.025 | 0.0783 | 0.745 | 0.042 | 0.89 | No |
| REN | 703 | -0.043 | 0.1234 | 0.728 | 0.039 | 0.89 | No |
| LEP | 704 | -0.026 | 0.0958 | 0.787 | 0.043 | 0.91 | No |
| PRL | 651 | 0.02 | 0.0801 | 0.802 | 0.044 | 0.92 | No |
| AGRP | 705 | 0.009 | 0.056 | 0.872 | 0.046 | 0.93 | No |
| CASP-8 | 542 | 0.014 | 0.0675 | 0.832 | 0.044 | 0.93 | No |
| EGF | 705 | -0.019 | 0.1197 | 0.873 | 0.047 | 0.93 | No |
| RAGE | 705 | -0.012 | 0.0645 | 0.848 | 0.045 | 0.93 | No |
| TRAIL | 705 | -0.008 | 0.0413 | 0.853 | 0.046 | 0.93 | No |
| PDGB Subunit B | 693 | 0.008 | 0.1039 | 0.939 | 0.048 | 0.98 | No |
| CCL20 | 705 | 0.001 | 0.1236 | 0.995 | 0.049 | 0.99 | No |
| IL-6RA | 705 | -0.001 | 0.0467 | 0.977 | 0.049 | 0.99 | No |
| SELE | 705 | 0.00005102 | 0.0792 | 0.999 | 0.050 | 0.99 | No |

Adjusted for age, age^2^, smoking status, body mass index, LDL-C, hsCRP, troponin T, diabetes, hypertension, myocardial infarction, coronary artery disease, number of NPX values below detection across all proteins

**Supplemental table 14**

**SNPs included in the weighted genetic risk score (wGRS) for myocardial fibrosis as previously reported (1)**

| **Chr** | **rsID** | **Nearest gene** | **Beta as reported in (1)** | **Risk allele** | **MAF** |
| --- | --- | --- | --- | --- | --- |
| 6 | rs2627230 | *SLC2A12* | 0.05 | A>T | 0.38 |
| 6 | rs9457699 | *SOD2* | -0.05 | T>G | 0.33 |
| 9 | rs1576900 | *ADAMTSL1* | -0.05 | G>A | 0.30 |
| 20 | rs6120777 | *MYH7B* | -0.06 | G>A | 0.16 |
| 6 | rs115740542 | *H2BC4/HFE* | -0.08 | T>C | 0.05 |
| 22 | rs855791 | *TMPRSS6* | 0.04 | A>G | 0.60 |
| 4 | rs55754224 | *CAMK2D* | 0.05 | C>T | 0.28 |
| 4 | rs365843 | *VEGFC* | -0.04 | C>T | 0.39 |
| 9 | rs58774558 | *KANK1* | 0.08 | C>G | 0.04 |
| 3 | rs13097267 | *PPP2R3A* | -0.04 | T>A | 0.29 |
| 6 | rs199754787 | *PIM1* | -0.13 | A>AT | 0.03 |

**Supplemental table 15**

**Association between fibrosis wGRS and mortality**

| **Model** | **Fibrosis GRS** | **All-cause mortality** | | **Cardiovascular mortality** | |
| --- | --- | --- | --- | --- | --- |
|  |  | **HR (95% CI)** | **P** | **HR (95% CI)** | **P** |
| Model 1 | ≤0 | Reference | | Reference | |
|  | >0 | 1.06 (0.92-1.22) | 0.451 | 1.14 (0.95-1.36) | 0.159 |

Model 1: adjusted for age, smoking status, body mass index, LDL-C, hsCRP, troponin T, diabetes, hypertension, acute coronary syndrome, coronary artery disease.

**Supplemental table 16**

**Association between LOY and all-cause mortality according to fibrosis GRS**

| **Model** | **LOY** | **Fibrosis GRS ≤0** | | **Fibrosis GRS >0** | |
| --- | --- | --- | --- | --- | --- |
|  |  | **HR (95% CI)** | **P** | **HR (95% CI)** | **P** |
| Model 1 | ≤17 % | Reference | | Reference | |
|  | >17 % | 1.16 (0.82-1.65) | 0.395 | 1.67 (1.08-2.59) | 0.007 |
| Model 2 | ≤17 % | Reference | | Reference | |
|  | >17 % | 1.27 (0.89-1.82) | 0.185 | 1.81 (1.16-2.83) | 0.025 |

Model 1: adjusted for age, age^2^, diabetes, hypertension, smoking, hsCRP, genotyping principal components.

Model 2: adjusted for age, age^2^, smoking status, body mass index, LDL-C, hsCRP, troponin T, diabetes, hypertension, myocardial infarction, coronary artery disease, genotyping principal components.

P for interaction between LOY and fibrosis GRS 0.229

**Supplemental table 17**

**Association between LOY and cardiovascular mortality according to fibrosis GRS**

| **Model** | **LOY** | **Fibrosis GRS ≤0** | | **Fibrosis GRS >0** | |
| --- | --- | --- | --- | --- | --- |
|  |  | **SHR (95% CI)** | **P** | **SHR (95% CI)** | **P** |
| Model 1 | ≤17 % | Reference | | Reference | |
|  | >17 % | 1.02 (0.62-1.67) | 0.936 | 2.09 (1.31-3.33) | 0.002 |
| Model 2 | ≤17 % | Reference | | Reference | |
|  | >17 % | 1.02 (0.62-1.69) | 0.926 | 2.14 (1.34-3.43) | 0.001 |

Model 1: adjusted for age, age^2^, diabetes, hypertension, smoking, hsCRP, genotyping principal components.

Model 2: adjusted for age, age^2^, smoking status, body mass index, LDL-C, hsCRP, troponin T, diabetes, hypertension, myocardial infarction, coronary artery disease, genotyping principal components.

P for interaction between LOY and fibrosis GRS 0.014

**Supplemental table 18**

**Significantly differentially methylated genes according to LOY**

| **CpG site**  **Identifier** | **F statistics** | **P value** | **FDR** | **Adjusted P value*** | **Mean**  **Beta**  **LOY >17 %** | **Mean**  **Beta**  **LOY ≤17 %** | **Chr** | **Mapinfo** | **UCSC RefGene Name** |
| --- | --- | --- | --- | --- | --- | --- | --- | --- | --- |
| cg13149459 | 245.003068 | 6.48E-45 | 4.78E-39 | 4.81E-39 | 0.724 | 0.651 | 1 | 202425679 | PPP1R12B |
| cg00063654 | 242.937968 | 1.29E-44 | 4.78E-39 | 9.56E-39 | 0.713 | 0.648 | 3 | 16413077 | RFTN1 |
| cg15846562 | 226.477994 | 3.31E-42 | 8.20E-37 | 2.46E-36 | 0.598 | 0.506 | X | 3560397 | PRKX |
| cg10892585 | 201.054564 | 2.28E-38 | 3.39E-33 | 1.69E-32 | 0.711 | 0.652 | 7 | 64298680 | ZNF138 |
| cg02027844 | 162.58256 | 2.83E-32 | 3.50E-27 | 2.10E-26 | 0.741 | 0.685 | X | 6992947 | HDHD1 |
| cg09104915 | 135.380759 | 9.77E-28 | 7.26E-23 | 7.26E-22 | 0.644 | 0.575 | 11 | 70867222 | SHANK2 |
| cg01900690 | 134.230153 | 1.54E-27 | 1.04E-22 | 1.14E-21 | 0.734 | 0.681 | X | 9696321 | GPR143 |
| cg17612569 | 117.037827 | 1.47E-24 | 7.30E-20 | 1.10E-18 | 0.127 | 0.159 | 21 | 27107221 | ATP5J |
| cg04162727 | 103.054952 | 4.58E-22 | 1.79E-17 | 3.40E-16 | 0.863 | 0.841 | X | 53227777 | KDM5C |
| cg14691167 | 87.4300473 | 3.32E-19 | 9.81E-15 | 2.47E-13 | 0.786 | 0.739 | X | 8432138 | VCX3B |
| cg13234275 | 85.6657317 | 7.07E-19 | 1.92E-14 | 5.25E-13 | 0.942 | 0.956 | 11 | 608991 | PHRF1 |
| cg22457256 | 85.6056641 | 7.26E-19 | 1.92E-14 | 5.39E-13 | 0.726 | 0.684 | 7 | 99574864 | AZGP1 |
| cg06513015 | 84.9113194 | 9.78E-19 | 2.42E-14 | 7.26E-13 | 0.691 | 0.652 | 7 | 64459246 | ERV3-1 |
| cg17054691 | 72.7521294 | 1.93E-16 | 3.99E-12 | 1.44E-10 | 0.845 | 0.882 | 17 | 79813439 | P4HB |
| cg14023774 | 68.7550355 | 1.13E-15 | 2.21E-11 | 8.39E-10 | 0.600 | 0.634 | 6 | 33246157 | B3GALT4 |
| cg05481201 | 68.1739912 | 1.46E-15 | 2.78E-11 | 1.09E-09 | 0.713 | 0.770 | 7 | 1585452 | TMEM184A |
| cg11831182 | 60.4419535 | 4.66E-14 | 7.68E-10 | 3.46E-08 | 0.814 | 0.840 | 1 | 156104614 | LMNA |
| cg25141766 | 59.6382702 | 6.69E-14 | 1.08E-09 | 4.97E-08 | 0.888 | 0.914 | 17 | 79826316 | ARHGDIA |
| cg17188688 | 59.4773667 | 7.20E-14 | 1.14E-09 | 5.34E-08 | 0.833 | 0.859 | 22 | 22333247 | TOP3B |
| cg24199203 | 57.5875343 | 1.69E-13 | 2.47E-09 | 1.26E-07 | 0.681 | 0.739 | 19 | 3387811 | NFIC |
| cg09857324 | 56.2993592 | 3.04E-13 | 4.18E-09 | 2.26E-07 | 0.737 | 0.782 | 16 | 1545151 | TELO2 |
| cg17636309 | 54.1628539 | 8.07E-13 | 1.02E-08 | 5.99E-07 | 0.820 | 0.846 | 8 | 144672242 | EEF1D |
| cg09911083 | 53.8489036 | 9.31E-13 | 1.15E-08 | 6.91E-07 | 0.543 | 0.566 | 17 | 7754956 | KDM6B |
| cg02716779 | 53.4419312 | 1.12E-12 | 1.37E-08 | 8.33E-07 | 0.626 | 0.595 | 9 | 131016143 | DNM1 |
| cg24087497 | 53.3990661 | 1.14E-12 | 1.37E-08 | 8.49E-07 | 0.680 | 0.710 | 12 | 54686330 | NFE2 |
| cg01819995 | 52.473372 | 1.75E-12 | 2.03E-08 | 1.30E-06 | 0.788 | 0.827 | 20 | 30537979 | PDRG1 |
| cg13707005 | 51.9488931 | 2.23E-12 | 2.54E-08 | 1.65E-06 | 0.750 | 0.780 | 10 | 11246628 | CUGBP2 |
| cg00199027 | 51.6911717 | 2.51E-12 | 2.79E-08 | 1.86E-06 | 0.699 | 0.738 | 17 | 1040002 | ABR |
| cg20551922 | 51.6828727 | 2.51E-12 | 2.79E-08 | 1.87E-06 | 0.710 | 0.747 | 17 | 4391282 | SPNS3 |
| cg00187704 | 51.1774463 | 3.17E-12 | 3.41E-08 | 2.36E-06 | 0.335 | 0.301 | 5 | 146131753 | PPP2R2B |
| cg17692096 | 51.0585941 | 3.35E-12 | 3.56E-08 | 2.49E-06 | 0.633 | 0.676 | 8 | 97773715 | CPQ |
| cg06340161 | 50.6826087 | 3.99E-12 | 4.11E-08 | 2.96E-06 | 0.759 | 0.792 | 16 | 46962962 | GPT2 |
| cg26364091 | 50.1802588 | 5.02E-12 | 5.11E-08 | 3.73E-06 | 0.594 | 0.627 | 22 | 41633219 | CHADL |
| cg11701312 | 49.1033217 | 8.26E-12 | 8.18E-08 | 6.13E-06 | 0.493 | 0.525 | 19 | 58897497 | RPS5 |
| cg14145074 | 48.1197799 | 1.30E-11 | 1.22E-07 | 9.67E-06 | 0.318 | 0.343 | 7 | 5567891 | ACTB |
| cg06033764 | 47.3506282 | 1.86E-11 | 1.69E-07 | 1.38E-05 | 0.790 | 0.820 | 18 | 29671448 | RNF138 |
| cg22128918 | 47.3496674 | 1.86E-11 | 1.69E-07 | 1.38E-05 | 0.725 | 0.759 | 6 | 31869088 | ZBTB12 |
| cg08087541 | 47.1046275 | 2.09E-11 | 1.82E-07 | 1.55E-05 | 0.512 | 0.536 | 11 | 837257 | CD151 |
| cg17821750 | 46.7068722 | 2.51E-11 | 2.14E-07 | 1.86E-05 | 0.932 | 0.948 | 19 | 811134 | PTBP1 |
| cg23665216 | 46.5986191 | 2.64E-11 | 2.22E-07 | 1.96E-05 | 0.791 | 0.817 | 14 | 105916422 | MTA1 |
| cg24718722 | 46.5788343 | 2.66E-11 | 2.22E-07 | 1.98E-05 | 0.481 | 0.591 | 10 | 129536818 | FOXI2 |
| cg01258042 | 46.5022622 | 2.76E-11 | 2.28E-07 | 2.05E-05 | 0.680 | 0.712 | 11 | 72416132 | ARAP1 |
| cg12806313 | 46.3369706 | 2.98E-11 | 2.39E-07 | 2.21E-05 | 0.432 | 0.476 | 19 | 33142994 | ANKRD27 |
| cg05083128 | 46.3302529 | 2.99E-11 | 2.39E-07 | 2.22E-05 | 0.639 | 0.699 | 4 | 10077759 | WDR1 |
| cg18570724 | 46.2758813 | 3.07E-11 | 2.42E-07 | 2.28E-05 | 0.416 | 0.392 | 4 | 7601257 | SORCS2 |
| cg09697874 | 46.039242 | 3.42E-11 | 2.65E-07 | 2.54E-05 | 0.647 | 0.677 | 19 | 45783658 | MARK4 |
| cg12492496 | 45.9413196 | 3.58E-11 | 2.74E-07 | 2.66E-05 | 0.725 | 0.774 | 19 | 18271859 | PIK3R2 |
| cg01554474 | 45.792565 | 3.84E-11 | 2.91E-07 | 2.85E-05 | 0.209 | 0.253 | 1 | 155107599 | RAG1AP1 |
| cg20010135 | 45.7215835 | 3.97E-11 | 2.98E-07 | 2.95E-05 | 0.567 | 0.628 | 16 | 30996822 | HSD3B7 |
| cg09371559 | 45.6964027 | 4.02E-11 | 2.98E-07 | 2.98E-05 | 0.656 | 0.688 | 19 | 17351300 | NR2F6 |
| cg18997635 | 45.6579326 | 4.09E-11 | 3.01E-07 | 3.04E-05 | 0.720 | 0.749 | 2 | 242449083 | STK25 |
| cg26642585 | 45.3931312 | 4.63E-11 | 3.28E-07 | 3.44E-05 | 0.584 | 0.609 | 3 | 183961869 | ALG3 |
| cg10007927 | 45.3721178 | 4.67E-11 | 3.28E-07 | 3.47E-05 | 0.621 | 0.646 | 6 | 32095148 | ATF6B |
| cg00787180 | 45.3706051 | 4.68E-11 | 3.28E-07 | 3.47E-05 | 0.620 | 0.681 | 14 | 91751731 | CCDC88C |
| cg06907892 | 45.0176917 | 5.51E-11 | 3.62E-07 | 4.09E-05 | 0.677 | 0.752 | Y | 14532343 | GYG2P1 |
| cg11297046 | 44.9765913 | 5.62E-11 | 3.66E-07 | 4.17E-05 | 0.491 | 0.520 | 19 | 49657000 | HRC |
| cg07020967 | 44.6801716 | 6.46E-11 | 4.13E-07 | 4.79E-05 | 0.595 | 0.638 | 12 | 44410498 | TMEM117 |
| cg23097139 | 44.5971383 | 6.71E-11 | 4.26E-07 | 4.98E-05 | 0.582 | 0.624 | 15 | 75918757 | SNUPN |
| cg05460965 | 44.4073929 | 7.33E-11 | 4.54E-07 | 5.44E-05 | 0.518 | 0.552 | 6 | 36645100 | CDKN1A |
| cg12599628 | 44.2547743 | 7.88E-11 | 4.79E-07 | 5.85E-05 | 0.784 | 0.824 | 7 | 22391521 | RAPGEF5 |
| cg11952344 | 44.119966 | 8.39E-11 | 5.06E-07 | 6.23E-05 | 0.681 | 0.716 | 17 | 80398854 | HEXDC |
| cg10100767 | 44.0402674 | 8.71E-11 | 5.19E-07 | 6.46E-05 | 0.846 | 0.870 | 14 | 105246561 | AKT1 |
| cg11575089 | 44.0324529 | 8.74E-11 | 5.19E-07 | 6.49E-05 | 0.308 | 0.348 | 1 | 155107462 | SLC50A1 |
| cg22904406 | 43.8851481 | 9.36E-11 | 5.46E-07 | 6.95E-05 | 0.629 | 0.663 | 6 | 33288296 | DAXX |
| cg02072975 | 43.7156591 | 1.01E-10 | 5.78E-07 | 7.53E-05 | 0.581 | 0.620 | 1 | 45249541 | BEST4 |
| cg14326472 | 43.6700148 | 1.04E-10 | 5.78E-07 | 7.69E-05 | 0.873 | 0.893 | 9 | 126164083 | DENND1A |
| cg12211691 | 43.1510784 | 1.32E-10 | 7.27E-07 | 9.80E-05 | 0.566 | 0.621 | 1 | 32211779 | ADGRB2 |
| cg05638011 | 43.0037151 | 1.42E-10 | 7.61E-07 | 0.00010506 | 0.327 | 0.343 | 11 | 73691625 | UCP2 |
| cg27560292 | 42.9017006 | 1.48E-10 | 7.87E-07 | 0.00011021 | 0.368 | 0.397 | 11 | 6632884 | TAF10 |
| cg10053073 | 42.8514222 | 1.52E-10 | 7.99E-07 | 0.00011284 | 0.518 | 0.545 | 17 | 73623182 | RECQL5 |
| cg14124980 | 42.8393915 | 1.53E-10 | 7.99E-07 | 0.00011348 | 0.557 | 0.581 | 11 | 66405450 | RBM4 |
| cg19431495 | 42.5473305 | 1.75E-10 | 8.92E-07 | 0.00013016 | 0.539 | 0.554 | 5 | 454456 | EXOC3 |
| cg07040244 | 42.2253113 | 2.04E-10 | 1.02E-06 | 0.00015141 | 0.331 | 0.369 | 1 | 231555522 | EGLN1 |
| cg03340102 | 41.9958951 | 2.27E-10 | 1.12E-06 | 0.00016865 | 0.394 | 0.419 | 10 | 22028443 | MLLT10 |
| cg07893565 | 41.7229851 | 2.58E-10 | 1.26E-06 | 0.00019175 | 0.577 | 0.521 | 4 | 84218838 | HPSE |
| cg19794606 | 41.4881327 | 2.88E-10 | 1.38E-06 | 0.00021416 | 0.490 | 0.517 | 6 | 138611752 | ARFGEF3 |
| cg18328494 | 41.179472 | 3.34E-10 | 1.59E-06 | 0.00024767 | 0.541 | 0.569 | 4 | 15725705 | BST1 |
| cg15120283 | 40.9373374 | 3.74E-10 | 1.76E-06 | 0.00027761 | 0.458 | 0.490 | 12 | 56436932 | RPS26 |
| cg10230957 | 40.8823266 | 3.84E-10 | 1.79E-06 | 0.00028491 | 0.820 | 0.852 | 5 | 149112344 | MIR378A |
| cg04780035 | 40.6991502 | 4.18E-10 | 1.94E-06 | 0.00031061 | 0.626 | 0.666 | 10 | 35012614 | PARD3 |
| cg08219938 | 40.6885211 | 4.21E-10 | 1.94E-06 | 0.00031217 | 0.677 | 0.703 | 17 | 1908237 | RTN4RL1 |
| cg24015005 | 40.5248375 | 4.54E-10 | 2.04E-06 | 0.00033724 | 0.427 | 0.453 | 7 | 1574098 | MAFK |
| cg09011401 | 40.3890264 | 4.84E-10 | 2.17E-06 | 0.00035956 | 0.535 | 0.598 | 1 | 216770086 | ESRRG |
| cg16082989 | 40.2921583 | 5.07E-10 | 2.25E-06 | 0.00037638 | 0.540 | 0.571 | 19 | 42613682 | POU2F2 |
| cg14546415 | 40.1940806 | 5.31E-10 | 2.35E-06 | 0.00039423 | 0.460 | 0.486 | 7 | 2277069 | FTSJ2 |
| cg02400530 | 40.1410084 | 5.45E-10 | 2.39E-06 | 0.00040423 | 0.359 | 0.378 | 1 | 1710880 | NADK |
| cg24201793 | 40.1165506 | 5.51E-10 | 2.41E-06 | 0.00040893 | 0.589 | 0.677 | 2 | 9144764 | MBOAT2 |
| cg24326574 | 39.8064125 | 6.38E-10 | 2.72E-06 | 0.00047348 | 0.685 | 0.725 | X | 153713930 | UBL4A |
| cg06269419 | 39.7541519 | 6.54E-10 | 2.77E-06 | 0.00048532 | 0.665 | 0.715 | 17 | 77807201 | CBX4 |
| cg03285215 | 39.5038989 | 7.36E-10 | 3.09E-06 | 0.0005463 | 0.490 | 0.519 | 6 | 31499987 | DDX39B |
| cg15916331 | 39.4299006 | 7.62E-10 | 3.18E-06 | 0.00056576 | 0.594 | 0.628 | 3 | 107262751 | BBX |
| cg15106082 | 38.8715997 | 9.93E-10 | 4.10E-06 | 0.00073696 | 0.566 | 0.597 | 2 | 240146162 | HDAC4 |
| cg20299697 | 38.8236917 | 1.02E-09 | 4.17E-06 | 0.00075388 | 0.502 | 0.556 | 3 | 138069423 | MRAS |
| cg09977126 | 38.7248472 | 1.06E-09 | 4.34E-06 | 0.00079004 | 0.418 | 0.372 | 5 | 75599987 | SV2C |
| cg20795999 | 38.5465593 | 1.16E-09 | 4.67E-06 | 0.00085973 | 0.813 | 0.828 | 3 | 184074817 | CLCN2 |
| cg23931548 | 38.4076507 | 1.24E-09 | 4.96E-06 | 0.00091828 | 0.657 | 0.676 | 1 | 55453452 | TMEM61 |
| cg14592794 | 38.3896181 | 1.25E-09 | 4.97E-06 | 0.00092617 | 0.257 | 0.287 | 17 | 26700958 | SARM1 |
| cg08754067 | 38.322112 | 1.29E-09 | 5.09E-06 | 0.00095631 | 0.305 | 0.337 | 5 | 40833236 | SNORD72 |
| cg00441709 | 38.0585606 | 1.46E-09 | 5.68E-06 | 0.00108375 | 0.734 | 0.789 | 17 | 2528280 | PAFAH1B1 |
| cg07703979 | 37.879313 | 1.59E-09 | 6.15E-06 | 0.00118005 | 0.454 | 0.491 | 1 | 53579547 | SLC1A7 |
| cg22321683 | 37.7933059 | 1.66E-09 | 6.37E-06 | 0.00122926 | 0.686 | 0.729 | 20 | 9598816 | PAK7 |
| cg25247520 | 37.6200869 | 1.80E-09 | 6.86E-06 | 0.00133474 | 0.577 | 0.601 | 8 | 128808017 | MIR1204 |
| cg17333398 | 37.6069692 | 1.81E-09 | 6.86E-06 | 0.00134309 | 0.753 | 0.780 | 12 | 133073006 | FBRSL1 |
| cg21194066 | 37.604621 | 1.81E-09 | 6.86E-06 | 0.00134459 | 0.632 | 0.665 | 11 | 67052509 | ADRBK1 |
| cg12275410 | 37.5810913 | 1.83E-09 | 6.90E-06 | 0.00135971 | 0.717 | 0.745 | 10 | 135123239 | ZNF511 |
| cg03527802 | 37.5149315 | 1.89E-09 | 7.09E-06 | 0.00140316 | 0.469 | 0.501 | 16 | 29819931 | MAZ |
| cg11878045 | 37.4619654 | 1.94E-09 | 7.20E-06 | 0.00143894 | 0.718 | 0.760 | 11 | 2482536 | KCNQ1 |
| cg21788755 | 37.4207022 | 1.98E-09 | 7.30E-06 | 0.00146746 | 0.450 | 0.468 | 3 | 48508391 | TREX1 |
| cg26006097 | 37.3238747 | 2.07E-09 | 7.57E-06 | 0.00153662 | 0.748 | 0.767 | 16 | 50338108 | ADCY7 |
| cg15352612 | 37.304479 | 2.09E-09 | 7.60E-06 | 0.00155086 | 0.731 | 0.756 | 16 | 57548915 | CCDC102A |
| cg09530095 | 37.2456131 | 2.15E-09 | 7.78E-06 | 0.00159491 | 0.696 | 0.728 | X | 153034414 | PLXNB3 |
| cg04146041 | 37.204201 | 2.19E-09 | 7.90E-06 | 0.00162665 | 0.680 | 0.707 | 12 | 53853849 | PCBP2 |
| cg03789579 | 36.8995503 | 2.53E-09 | 8.91E-06 | 0.00188051 | 0.750 | 0.770 | 1 | 32670241 | CCDC28B |
| cg12499872 | 36.8575656 | 2.58E-09 | 9.01E-06 | 0.00191849 | 0.773 | 0.816 | 16 | 58019893 | TEPP |
| cg01419914 | 36.8478007 | 2.60E-09 | 9.01E-06 | 0.00192743 | 0.591 | 0.641 | 17 | 79374691 | BAHCC1 |
| cg19934294 | 36.7106665 | 2.77E-09 | 9.48E-06 | 0.00205754 | 0.408 | 0.429 | 4 | 2933421 | MFSD10 |
| cg24503449 | 36.6777031 | 2.82E-09 | 9.59E-06 | 0.00209011 | 0.756 | 0.779 | 19 | 18266888 | PIK3R2 |
| cg13810673 | 36.6577027 | 2.84E-09 | 9.60E-06 | 0.00211013 | 0.139 | 0.110 | 14 | 88473038 | GPR65 |
| cg24337975 | 36.6025334 | 2.92E-09 | 9.76E-06 | 0.00216633 | 0.673 | 0.707 | 2 | 233876260 | NGEF |
| cg21774980 | 36.5555581 | 2.98E-09 | 9.89E-06 | 0.00221538 | 0.238 | 0.254 | 20 | 34090548 | CEP250 |
| cg10829227 | 36.5506196 | 2.99E-09 | 9.89E-06 | 0.0022206 | 0.738 | 0.763 | 19 | 47200595 | PRKD2 |
| cg17246606 | 36.4861738 | 3.08E-09 | 1.01E-05 | 0.00228987 | 0.817 | 0.840 | 1 | 16266161 | SPEN |
| cg07773475 | 36.4737162 | 3.10E-09 | 1.01E-05 | 0.00230351 | 0.358 | 0.389 | 17 | 39741002 | KRT14 |
| cg24771793 | 36.2900561 | 3.39E-09 | 1.09E-05 | 0.00251435 | 0.493 | 0.526 | 6 | 43189132 | CUL9 |
| cg17775079 | 36.2586841 | 3.44E-09 | 1.10E-05 | 0.00255225 | 0.755 | 0.788 | 10 | 65224441 | JMJD1C |
| cg04190002 | 36.2480765 | 3.46E-09 | 1.10E-05 | 0.0025652 | 0.316 | 0.302 | 22 | 51113604 | SHANK3 |
| cg03043406 | 36.2476874 | 3.46E-09 | 1.10E-05 | 0.00256567 | 0.534 | 0.586 | 1 | 45242356 | RPS8 |
| cg06482315 | 36.1562654 | 3.61E-09 | 1.14E-05 | 0.00268005 | 0.694 | 0.733 | 20 | 49195860 | PTPN1 |
| cg20968821 | 36.147263 | 3.63E-09 | 1.14E-05 | 0.00269158 | 0.606 | 0.624 | 14 | 105522684 | GPR132 |
| cg08800277 | 36.0871365 | 3.73E-09 | 1.16E-05 | 0.00276992 | 0.660 | 0.689 | 2 | 96577476 | ANKRD36C |
| cg00753112 | 36.0283792 | 3.84E-09 | 1.18E-05 | 0.0028487 | 0.601 | 0.632 | 8 | 136593301 | KHDRBS3 |
| cg08625564 | 36.026651 | 3.84E-09 | 1.18E-05 | 0.00285105 | 0.690 | 0.716 | 17 | 1553453 | RILP |
| cg00127150 | 35.9819756 | 3.92E-09 | 1.20E-05 | 0.0029125 | 0.701 | 0.741 | 22 | 22862144 | ZNF280B |
| cg17894416 | 35.8590176 | 4.16E-09 | 1.27E-05 | 0.00308859 | 0.080 | 0.061 | 14 | 61809318 | PRKCH |
| cg04394939 | 35.786408 | 4.31E-09 | 1.30E-05 | 0.00319756 | 0.697 | 0.728 | 19 | 1212172 | STK11 |
| cg00014380 | 35.7516944 | 4.38E-09 | 1.32E-05 | 0.00325102 | 0.817 | 0.843 | 16 | 4424290 | VASN |
| cg20659941 | 35.7376193 | 4.41E-09 | 1.32E-05 | 0.00327294 | 0.668 | 0.693 | 18 | 55888395 | NEDD4L |
| cg13590876 | 35.6784092 | 4.54E-09 | 1.35E-05 | 0.00336684 | 0.629 | 0.668 | 14 | 105154689 | INF2 |
| cg21892407 | 35.6630188 | 4.57E-09 | 1.36E-05 | 0.00339168 | 0.358 | 0.317 | 2 | 166006239 | SCN3A |
| cg01966510 | 35.646697 | 4.61E-09 | 1.36E-05 | 0.00341823 | 0.694 | 0.647 | 15 | 85075427 | UBE2Q2P1 |
| cg16289349 | 35.5535622 | 4.81E-09 | 1.41E-05 | 0.00357379 | 0.632 | 0.682 | 3 | 46784830 | PRSS45 |
| cg01428071 | 35.3831313 | 5.22E-09 | 1.52E-05 | 0.00387712 | 0.494 | 0.528 | 6 | 79785773 | PHIP |
| cg13644239 | 35.3051001 | 5.42E-09 | 1.57E-05 | 0.00402451 | 0.519 | 0.545 | 5 | 891886 | TRIP13 |
| cg01935016 | 35.2514324 | 5.56E-09 | 1.59E-05 | 0.00412914 | 0.499 | 0.543 | X | 9866048 | SHROOM2 |
| cg08345719 | 35.2004197 | 5.70E-09 | 1.62E-05 | 0.00423112 | 0.804 | 0.837 | 17 | 37821661 | TCAP |
| cg07072176 | 35.1951122 | 5.71E-09 | 1.62E-05 | 0.00424188 | 0.777 | 0.800 | 8 | 140906190 | TRAPPC9 |
| cg26450266 | 35.1854587 | 5.74E-09 | 1.62E-05 | 0.0042615 | 0.605 | 0.634 | 17 | 18149687 | FLII |
| cg14694639 | 35.1264772 | 5.91E-09 | 1.65E-05 | 0.00438345 | 0.522 | 0.558 | 1 | 147039678 | BCL9 |
| cg03823989 | 35.0924026 | 6.00E-09 | 1.66E-05 | 0.00445549 | 0.507 | 0.538 | 9 | 100292179 | TMOD1 |
| cg26775538 | 35.0904976 | 6.01E-09 | 1.66E-05 | 0.00445955 | 0.600 | 0.642 | 19 | 815090 | LPPR3 |
| cg22140810 | 35.0751909 | 6.05E-09 | 1.67E-05 | 0.00449233 | 0.728 | 0.756 | 7 | 143056983 | FAM131B |
| cg08362880 | 34.8967682 | 6.59E-09 | 1.80E-05 | 0.0048928 | 0.447 | 0.481 | 6 | 32053600 | TNXB |
| cg15838394 | 34.8725521 | 6.67E-09 | 1.81E-05 | 0.00494985 | 0.492 | 0.519 | 11 | 73358930 | PLEKHB1 |
| cg07048256 | 34.7266326 | 7.15E-09 | 1.93E-05 | 0.00530809 | 0.559 | 0.581 | 14 | 24733374 | TGM1 |
| cg23519673 | 34.7251703 | 7.16E-09 | 1.93E-05 | 0.0053118 | 0.493 | 0.521 | 12 | 42979733 | PRICKLE1 |
| cg03422895 | 34.673071 | 7.34E-09 | 1.97E-05 | 0.00544602 | 0.609 | 0.639 | 17 | 2598389 | KIAA0664 |
| cg24951886 | 34.6594824 | 7.39E-09 | 1.97E-05 | 0.00548158 | 0.661 | 0.686 | 10 | 13526236 | BEND7 |
| cg17467146 | 34.604097 | 7.58E-09 | 2.00E-05 | 0.00562896 | 0.652 | 0.667 | 1 | 153723713 | INTS3 |
| cg08229518 | 34.5658224 | 7.72E-09 | 2.03E-05 | 0.00573313 | 0.588 | 0.608 | 4 | 1320300 | MAEA |
| cg01457573 | 34.5261904 | 7.87E-09 | 2.06E-05 | 0.00584305 | 0.531 | 0.555 | 11 | 72003887 | CLPB |
| cg11597157 | 34.5159758 | 7.91E-09 | 2.06E-05 | 0.00587171 | 0.890 | 0.908 | 15 | 90171643 | KIF7 |
| cg21370352 | 34.4818616 | 8.04E-09 | 2.09E-05 | 0.00596848 | 0.693 | 0.725 | 1 | 19651020 | PQLC2 |
| cg08907118 | 34.4697563 | 8.09E-09 | 2.09E-05 | 0.0060032 | 0.600 | 0.622 | 16 | 27482516 | GTF3C1 |
| cg27275941 | 34.4124164 | 8.31E-09 | 2.14E-05 | 0.00617047 | 0.589 | 0.562 | 3 | 15751015 | ANKRD28 |
| cg15081062 | 34.4003446 | 8.36E-09 | 2.14E-05 | 0.00620628 | 0.751 | 0.779 | 3 | 25832554 | OXSM |
| cg27165960 | 34.3366185 | 8.62E-09 | 2.19E-05 | 0.0063988 | 0.327 | 0.289 | 5 | 39177381 | FYB |
| cg09324457 | 34.3348398 | 8.63E-09 | 2.19E-05 | 0.00640425 | 0.583 | 0.618 | 19 | 41196008 | NUMBL |
| cg18353028 | 34.2549743 | 8.97E-09 | 2.26E-05 | 0.00665425 | 0.460 | 0.416 | 8 | 65669513 | CYP7B1 |
| cg13914910 | 34.2293275 | 9.08E-09 | 2.27E-05 | 0.00673659 | 0.496 | 0.532 | 10 | 72576682 | SGPL1 |
| cg05023526 | 34.1860691 | 9.27E-09 | 2.30E-05 | 0.0068778 | 0.486 | 0.540 | 4 | 41695014 | LIMCH1 |
| cg09970855 | 34.1744032 | 9.32E-09 | 2.31E-05 | 0.00691639 | 0.584 | 0.639 | 5 | 149112199 | MIR378A |
| cg07310916 | 34.1521353 | 9.42E-09 | 2.32E-05 | 0.00699065 | 0.580 | 0.603 | 2 | 232330273 | NCL |
| cg07912766 | 34.1465709 | 9.44E-09 | 2.32E-05 | 0.00700933 | 0.559 | 0.584 | 18 | 45458698 | SMAD2 |
| cg09944697 | 34.1424096 | 9.46E-09 | 2.32E-05 | 0.00702332 | 0.938 | 0.949 | 14 | 105685660 | BRF1 |
| cg14381448 | 34.108854 | 9.62E-09 | 2.35E-05 | 0.00713729 | 0.240 | 0.265 | 17 | 42336559 | SLC4A1 |
| cg11409350 | 33.9943733 | 1.02E-08 | 2.47E-05 | 0.00754031 | 0.793 | 0.827 | 17 | 73126499 | NT5C |
| cg12741900 | 33.9776015 | 1.02E-08 | 2.48E-05 | 0.00760124 | 0.764 | 0.789 | 1 | 155165786 | THBS3 |
| cg13862013 | 33.9718105 | 1.03E-08 | 2.48E-05 | 0.00762239 | 0.629 | 0.658 | 11 | 68549689 | CPT1A |
| cg15789106 | 33.8807117 | 1.07E-08 | 2.58E-05 | 0.00796307 | 0.421 | 0.442 | 11 | 563990 | RASSF7 |
| cg00483891 | 33.8622532 | 1.08E-08 | 2.59E-05 | 0.00803393 | 0.720 | 0.760 | 2 | 131098007 | CCDC115 |
| cg22997766 | 33.8516868 | 1.09E-08 | 2.60E-05 | 0.00807478 | 0.709 | 0.731 | 12 | 53456673 | TNS2 |
| cg03533141 | 33.8469794 | 1.09E-08 | 2.60E-05 | 0.00809304 | 0.300 | 0.327 | 1 | 45243518 | SNORD38A |
| cg02715602 | 33.8037494 | 1.11E-08 | 2.64E-05 | 0.00826276 | 0.814 | 0.860 | 19 | 4544446 | SEMA6B |
| cg02329699 | 33.762116 | 1.14E-08 | 2.69E-05 | 0.0084296 | 0.457 | 0.404 | 18 | 22814014 | ZNF521 |
| cg26846629 | 33.7516768 | 1.14E-08 | 2.69E-05 | 0.00847195 | 0.675 | 0.696 | 3 | 185948554 | DGKG |
| cg16589911 | 33.7068821 | 1.17E-08 | 2.73E-05 | 0.00865616 | 0.577 | 0.611 | 1 | 161184489 | FCER1G |
| cg05417607 | 33.6840992 | 1.18E-08 | 2.75E-05 | 0.00875139 | 0.921 | 0.951 | 17 | 1373605 | MYO1C |
| cg26385126 | 33.6453848 | 1.20E-08 | 2.79E-05 | 0.00891563 | 0.767 | 0.797 | 12 | 124912021 | NCOR2 |
| cg05753470 | 33.5309549 | 1.27E-08 | 2.93E-05 | 0.00941948 | 0.544 | 0.531 | 19 | 33187374 | NUDT19 |
| cg04273431 | 33.5207259 | 1.28E-08 | 2.93E-05 | 0.00946588 | 0.586 | 0.625 | 6 | 30523215 | GNL1 |
| cg09104072 | 33.4740155 | 1.30E-08 | 2.98E-05 | 0.00968073 | 0.733 | 0.748 | 11 | 31418445 | DNAJC24 |
| cg07728212 | 33.4446047 | 1.32E-08 | 3.00E-05 | 0.00981852 | 0.536 | 0.559 | 22 | 32022290 | PISD |
| cg16392086 | 33.4213366 | 1.34E-08 | 3.03E-05 | 0.00992893 | 0.382 | 0.410 | 11 | 62444389 | UBXN1 |
| cg11852940 | 33.390555 | 1.36E-08 | 3.06E-05 | 0.01007691 | 0.887 | 0.900 | 11 | 65364071 | KCNK7 |
| cg14703138 | 33.2640939 | 1.44E-08 | 3.24E-05 | 0.01070857 | 0.538 | 0.556 | 7 | 128552004 | KCP;KCP |
| cg03011059 | 33.2586529 | 1.45E-08 | 3.24E-05 | 0.01073661 | 0.596 | 0.640 | 12 | 120935568 | DYNLL1 |
| cg10550302 | 33.2424236 | 1.46E-08 | 3.24E-05 | 0.0108207 | 0.650 | 0.621 | 5 | 31995926 | PDZD2 |
| cg05895403 | 33.2337721 | 1.46E-08 | 3.24E-05 | 0.0108658 | 0.609 | 0.631 | 16 | 31150380 | PRSS36 |
| cg04486013 | 33.2210481 | 1.47E-08 | 3.25E-05 | 0.01093248 | 0.592 | 0.624 | 1 | 46035287 | AKR1A1 |
| cg14334382 | 33.1949037 | 1.49E-08 | 3.25E-05 | 0.01107076 | 0.468 | 0.496 | 7 | 27703802 | HIBADH |
| cg04544154 | 33.1375572 | 1.53E-08 | 3.31E-05 | 0.0113803 | 0.766 | 0.800 | X | 129245509 | ELF4 |
| cg03918288 | 33.0964088 | 1.56E-08 | 3.36E-05 | 0.01160779 | 0.553 | 0.584 | 8 | 145101324 | SPATC1 |
| cg19857714 | 33.0718765 | 1.58E-08 | 3.37E-05 | 0.01174557 | 0.617 | 0.649 | 11 | 986709 | AP2A2 |
| cg03823477 | 33.0677348 | 1.59E-08 | 3.37E-05 | 0.01176898 | 0.343 | 0.328 | 7 | 34764107 | NPSR1 |
| cg17991341 | 33.0140465 | 1.63E-08 | 3.45E-05 | 0.01207695 | 0.550 | 0.576 | 1 | 228296128 | MRPL55 |
| cg01997290 | 32.9788906 | 1.65E-08 | 3.50E-05 | 0.01228298 | 0.835 | 0.862 | 22 | 48970763 | FAM19A5 |
| cg00217953 | 32.9698506 | 1.66E-08 | 3.51E-05 | 0.01233652 | 0.609 | 0.654 | 2 | 97527635 | SEMA4C |
| cg25818109 | 32.9195595 | 1.70E-08 | 3.56E-05 | 0.01263871 | 0.555 | 0.527 | 3 | 186937654 | MASP1 |
| cg09240391 | 32.8984729 | 1.72E-08 | 3.59E-05 | 0.01276762 | 0.488 | 0.511 | 19 | 54962416 | LENG8 |
| cg01475205 | 32.8661873 | 1.75E-08 | 3.63E-05 | 0.01296758 | 0.476 | 0.508 | 11 | 64412026 | NRXN2 |
| cg25867318 | 32.8330378 | 1.78E-08 | 3.68E-05 | 0.01317617 | 0.508 | 0.454 | 17 | 40494745 | STAT3 |
| cg05711037 | 32.7910342 | 1.81E-08 | 3.72E-05 | 0.01344529 | 0.726 | 0.757 | 2 | 234359783 | DGKD |
| cg04051044 | 32.7901505 | 1.81E-08 | 3.72E-05 | 0.01345099 | 0.524 | 0.546 | 21 | 47666523 | MCM3AP |
| cg24550676 | 32.7331722 | 1.86E-08 | 3.80E-05 | 0.01382511 | 0.708 | 0.730 | 5 | 140053903 | DND1 |
| cg00502254 | 32.6917709 | 1.90E-08 | 3.87E-05 | 0.01410352 | 0.638 | 0.668 | 1 | 12201600 | TNFRSF8 |
| cg01753544 | 32.6556063 | 1.93E-08 | 3.90E-05 | 0.01435129 | 0.473 | 0.496 | 11 | 64509592 | RASGRP2 |
| cg22799840 | 32.6553056 | 1.93E-08 | 3.90E-05 | 0.01435335 | 0.825 | 0.863 | 22 | 41908900 | ACO2 |
| cg13798109 | 32.6480013 | 1.94E-08 | 3.90E-05 | 0.01440392 | 0.625 | 0.665 | 5 | 176396084 | UIMC1 |
| cg25976340 | 32.6369375 | 1.95E-08 | 3.90E-05 | 0.01448087 | 0.532 | 0.549 | 11 | 18611549 | UEVLD |
| cg09788082 | 32.6319014 | 1.96E-08 | 3.90E-05 | 0.014516 | 0.688 | 0.716 | 19 | 1163485 | SBNO2 |
| cg22101249 | 32.6045233 | 1.98E-08 | 3.93E-05 | 0.01470867 | 0.712 | 0.739 | 6 | 32131615 | EGFL8 |
| cg24752967 | 32.5983446 | 1.99E-08 | 3.94E-05 | 0.0147525 | 0.355 | 0.388 | 8 | 22411728 | SORBS3 |
| cg13492133 | 32.5815874 | 2.00E-08 | 3.95E-05 | 0.01487206 | 0.825 | 0.843 | 1 | 183516266 | SMG7 |
| cg08252353 | 32.5804294 | 2.01E-08 | 3.95E-05 | 0.01488034 | 0.665 | 0.681 | 19 | 36506567 | ALKBH6 |
| cg04169221 | 32.5183333 | 2.07E-08 | 4.05E-05 | 0.01533222 | 0.492 | 0.529 | 6 | 135507123 | MYB |
| cg05614191 | 32.513228 | 2.07E-08 | 4.05E-05 | 0.01536996 | 0.897 | 0.917 | 5 | 115171433 | ATG12 |
| cg08657391 | 32.4235428 | 2.16E-08 | 4.20E-05 | 0.01604879 | 0.488 | 0.511 | 7 | 72768697 | FKBP6 |
| cg09323400 | 32.3995253 | 2.19E-08 | 4.24E-05 | 0.01623564 | 0.796 | 0.817 | 12 | 113535362 | DTX1 |
| cg20494891 | 32.3161964 | 2.28E-08 | 4.33E-05 | 0.01690099 | 0.382 | 0.347 | 15 | 59642565 | MYO1E |
| cg12179661 | 32.315058 | 2.28E-08 | 4.33E-05 | 0.01691024 | 0.477 | 0.511 | 9 | 140333805 | ENTPD8 |
| cg14562086 | 32.312255 | 2.28E-08 | 4.33E-05 | 0.01693306 | 0.299 | 0.325 | 17 | 48279265 | COL1A1 |
| cg24496349 | 32.3041555 | 2.29E-08 | 4.34E-05 | 0.01699929 | 0.868 | 0.883 | 11 | 67810905 | TCIRG1 |
| cg01839993 | 32.2760615 | 2.32E-08 | 4.36E-05 | 0.01723106 | 0.343 | 0.372 | 10 | 74034644 | DDIT4 |
| cg10493259 | 32.2666614 | 2.33E-08 | 4.37E-05 | 0.01730932 | 0.728 | 0.760 | 7 | 151092290 | WDR86 |
| cg08643012 | 32.1331303 | 2.49E-08 | 4.61E-05 | 0.01846058 | 0.736 | 0.761 | 13 | 37494946 | SMAD9 |
| cg11415341 | 32.1072293 | 2.52E-08 | 4.65E-05 | 0.01869265 | 0.855 | 0.876 | 17 | 27418879 | MYO18A |
| cg25550013 | 32.0857886 | 2.54E-08 | 4.68E-05 | 0.01888695 | 0.558 | 0.576 | 16 | 56915638 | SLC12A3 |
| cg11621113 | 32.0093968 | 2.64E-08 | 4.75E-05 | 0.01959586 | 0.270 | 0.300 | 19 | 12776725 | MORG1 |
| cg26771969 | 31.9957762 | 2.66E-08 | 4.77E-05 | 0.01972503 | 0.799 | 0.819 | 19 | 15358258 | BRD4 |
| cg05008895 | 31.89394 | 2.79E-08 | 4.96E-05 | 0.02071859 | 0.260 | 0.286 | 16 | 1458735 | UNKL |
| cg13686739 | 31.889092 | 2.80E-08 | 4.96E-05 | 0.0207671 | 0.794 | 0.815 | 17 | 48211226 | PPP1R9B |
| cg07260508 | 31.8816432 | 2.81E-08 | 4.97E-05 | 0.02084188 | 0.503 | 0.560 | 11 | 131799134 | NTM |
| cg09489844 | 31.8434899 | 2.86E-08 | 5.03E-05 | 0.02122924 | 0.944 | 0.956 | 17 | 79880647 | MAFG |
| cg01303141 | 31.8139874 | 2.90E-08 | 5.09E-05 | 0.02153375 | 0.795 | 0.827 | 17 | 77952109 | TBC1D16 |
| cg17473615 | 31.802939 | 2.92E-08 | 5.11E-05 | 0.0216489 | 0.497 | 0.532 | X | 54835651 | MAGED2 |
| cg26559055 | 31.7945171 | 2.93E-08 | 5.12E-05 | 0.02173708 | 0.786 | 0.806 | Y | 21154291 | TTTY14 |
| cg00233018 | 31.7784521 | 2.95E-08 | 5.15E-05 | 0.02190631 | 0.531 | 0.557 | 4 | 2943520 | NOP14 |
| cg02040650 | 31.6014787 | 3.22E-08 | 5.57E-05 | 0.02386096 | 0.670 | 0.696 | 20 | 57604021 | ATP5E |
| cg05860102 | 31.5590077 | 3.28E-08 | 5.67E-05 | 0.02435558 | 0.812 | 0.793 | 15 | 101088876 | PRKXP1 |
| cg20287434 | 31.5318682 | 3.33E-08 | 5.73E-05 | 0.02467702 | 0.707 | 0.740 | 17 | 79958592 | ASPSCR1 |
| cg12303084 | 31.4121705 | 3.52E-08 | 6.04E-05 | 0.02614652 | 0.175 | 0.201 | 20 | 45985741 | ZMYND8 |
| cg11231913 | 31.3937999 | 3.55E-08 | 6.07E-05 | 0.02637969 | 0.680 | 0.701 | 20 | 33577529 | MIR499 |
| cg00966405 | 31.3764903 | 3.58E-08 | 6.09E-05 | 0.02660123 | 0.776 | 0.795 | 7 | 5535748 | MIR589 |
| cg15112783 | 31.309028 | 3.70E-08 | 6.22E-05 | 0.02748296 | 0.802 | 0.819 | 20 | 33147614 | MAP1LC3A |
| cg21288249 | 31.3068438 | 3.71E-08 | 6.22E-05 | 0.02751197 | 0.822 | 0.839 | 16 | 69961491 | WWP2 |
| cg09036263 | 31.2832315 | 3.75E-08 | 6.27E-05 | 0.02782779 | 0.700 | 0.716 | 1 | 6154349 | KCNAB2 |
| cg12884495 | 31.2767292 | 3.76E-08 | 6.28E-05 | 0.02791538 | 0.611 | 0.639 | 17 | 73842017 | WBP2 |
| cg15619425 | 31.2661007 | 3.78E-08 | 6.30E-05 | 0.02805918 | 0.629 | 0.652 | 9 | 133977887 | AIF1L |
| cg11277126 | 31.2170601 | 3.87E-08 | 6.39E-05 | 0.02873244 | 0.373 | 0.398 | 20 | 33680945 | TRPC4AP |
| cg10788371 | 31.2125462 | 3.88E-08 | 6.39E-05 | 0.02879516 | 0.496 | 0.538 | 11 | 76381040 | LRRC32 |
| cg20954129 | 31.187205 | 3.93E-08 | 6.44E-05 | 0.02915021 | 0.709 | 0.728 | 6 | 33259866 | RGL2;RGL2 |
| cg00672192 | 31.1861885 | 3.93E-08 | 6.44E-05 | 0.0291645 | 0.618 | 0.648 | 17 | 76073640 | TNRC6C |
| cg01997461 | 31.134883 | 4.03E-08 | 6.57E-05 | 0.02989723 | 0.628 | 0.675 | 13 | 98825992 | FARP1 |
| cg17400925 | 31.09675 | 4.10E-08 | 6.67E-05 | 0.03045382 | 0.612 | 0.622 | 1 | 228332329 | GUK1 |
| cg07184986 | 31.0752615 | 4.15E-08 | 6.72E-05 | 0.03077199 | 0.692 | 0.713 | 5 | 159714788 | CCNJL |
| cg12652118 | 31.0620861 | 4.17E-08 | 6.75E-05 | 0.03096874 | 0.338 | 0.354 | 8 | 22858539 | RHOBTB2 |
| cg13796737 | 31.0386061 | 4.22E-08 | 6.78E-05 | 0.03132244 | 0.564 | 0.583 | 22 | 31968954 | SFI1 |
| cg08662753 | 30.982146 | 4.34E-08 | 6.94E-05 | 0.03218992 | 0.518 | 0.559 | 9 | 137561500 | COL5A1 |
| cg23145355 | 30.9708601 | 4.36E-08 | 6.96E-05 | 0.03236617 | 0.838 | 0.848 | 6 | 10622183 | GCNT2 |
| cg01215511 | 30.9645379 | 4.37E-08 | 6.96E-05 | 0.03246527 | 0.701 | 0.760 | 16 | 2975552 | FLYWCH1 |
| cg08685909 | 30.9502177 | 4.41E-08 | 6.99E-05 | 0.03269101 | 0.401 | 0.422 | 20 | 57559409 | TH1L |
| cg11082684 | 30.9400337 | 4.43E-08 | 7.01E-05 | 0.0328525 | 0.332 | 0.350 | 7 | 98591756 | TRRAP |
| cg17783244 | 30.9052407 | 4.50E-08 | 7.09E-05 | 0.03341035 | 0.626 | 0.658 | 22 | 31738549 | PATZ1 |
| cg00553487 | 30.901998 | 4.51E-08 | 7.09E-05 | 0.03346279 | 0.554 | 0.594 | 19 | 42570406 | GRIK5 |
| cg09795027 | 30.8632979 | 4.59E-08 | 7.17E-05 | 0.0340954 | 0.423 | 0.456 | 17 | 74006440 | EVPL |
| cg12728032 | 30.8259794 | 4.68E-08 | 7.27E-05 | 0.03471691 | 0.774 | 0.792 | 11 | 34535357 | ELF5 |
| cg08165261 | 30.8003126 | 4.74E-08 | 7.33E-05 | 0.03515094 | 0.787 | 0.805 | 19 | 18977240 | UPF1 |
| cg00815325 | 30.7644831 | 4.82E-08 | 7.44E-05 | 0.03576606 | 0.463 | 0.491 | 19 | 5691899 | LONP1 |
| cg01325154 | 30.7557469 | 4.84E-08 | 7.44E-05 | 0.03591764 | 0.792 | 0.809 | 17 | 48823123 | LUC7L3 |
| cg26361535 | 30.7553384 | 4.84E-08 | 7.44E-05 | 0.0359247 | 0.662 | 0.722 | 8 | 144576604 | ZC3H3 |
| cg16282569 | 30.7368932 | 4.88E-08 | 7.48E-05 | 0.03624698 | 0.564 | 0.581 | 17 | 79658055 | HGS |
| cg03169180 | 30.7215103 | 4.92E-08 | 7.52E-05 | 0.03651793 | 0.625 | 0.650 | 17 | 7310347 | NLGN2 |
| cg04486136 | 30.6840561 | 5.01E-08 | 7.63E-05 | 0.03718631 | 0.768 | 0.792 | 19 | 52702171 | PPP2R1A |
| cg05689068 | 30.5149786 | 5.44E-08 | 8.19E-05 | 0.04036034 | 0.792 | 0.807 | 1 | 11889823 | CLCN6 |
| cg04644846 | 30.4892843 | 5.51E-08 | 8.25E-05 | 0.04086593 | 0.667 | 0.690 | 22 | 51012969 | CPT1B |
| cg12283875 | 30.4754439 | 5.54E-08 | 8.28E-05 | 0.04114085 | 0.628 | 0.651 | 15 | 59734208 | FAM81A |
| cg01845041 | 30.4512694 | 5.61E-08 | 8.35E-05 | 0.0416256 | 0.437 | 0.477 | 3 | 118753005 | IGSF11 |
| cg04201752 | 30.324561 | 5.97E-08 | 8.82E-05 | 0.0442624 | 0.521 | 0.552 | 3 | 50360397 | HYAL2 |
| cg00402516 | 30.2755514 | 6.11E-08 | 9.00E-05 | 0.04532681 | 0.636 | 0.659 | 19 | 1580504 | MBD3 |
| cg07560588 | 30.2746982 | 6.11E-08 | 9.00E-05 | 0.04534551 | 0.423 | 0.456 | 1 | 67884788 | SERBP1 |
| cg05525364 | 30.2413521 | 6.21E-08 | 9.13E-05 | 0.0460847 | 0.602 | 0.632 | X | 64741336 | LAS1L |
| cg26073542 | 30.2269027 | 6.25E-08 | 9.18E-05 | 0.04640872 | 0.761 | 0.784 | 11 | 3174128 | OSBPL5 |
| cg26066277 | 30.1897754 | 6.37E-08 | 9.31E-05 | 0.04725187 | 0.921 | 0.939 | 17 | 79792777 | DYSFIP1 |
| cg05661982 | 30.1857077 | 6.38E-08 | 9.31E-05 | 0.04734513 | 0.789 | 0.817 | 7 | 2420084 | EIF3B |
| cg09075063 | 30.1479729 | 6.50E-08 | 9.46E-05 | 0.04821967 | 0.176 | 0.201 | X | 40036659 | BCOR |
| cg08398184 | 30.1072105 | 6.63E-08 | 9.63E-05 | 0.04918266 | 0.694 | 0.725 | 9 | 131755527 | NUP188 |
| cg05017552 | 30.0949404 | 6.67E-08 | 9.65E-05 | 0.04947619 | 0.811 | 0.825 | 18 | 72187476 | CNDP2;CNDP2 |
| cg13180508 | 30.0820526 | 6.71E-08 | 9.69E-05 | 0.04978646 | 0.500 | 0.518 | 22 | 24179356 | DERL3 |

* by Holm-Bonferroni method

**Supplemental table 19**

**Gene expression of all differentially methylated genes in scRNAseq analyses of Y-positive and LOY leukocytes**

| **Gene** | **P value (unadjusted)** | **Log fold-change of the average expression between both groups (Y-positive cells as reference)** | **Adjusted P value*** |
| --- | --- | --- | --- |
| RPS8 | 3.986E-246 | -0.4866969 | 8.38E-242 |
| RPS5 | 1.099E-240 | -0.5935121 | 2.31E-236 |
| FCER1G | 3.656E-200 | 0.84855549 | 7.686E-196 |
| RPS26 | 3.675E-155 | -0.5922979 | 7.726E-151 |
| ACTB | 3.855E-102 | 0.43523102 | 8.1055E-98 |
| EEF1D | 4.5784E-47 | -0.2007931 | 9.6256E-43 |
| TCIRG1 | 3.012E-41 | 0.68671482 | 6.3325E-37 |
| DYNLL1 | 4.0383E-28 | 0.52863319 | 8.4901E-24 |
| CCNJL | 1.4522E-27 | 1.88625624 | 3.053E-23 |
| ATP5F1E | 5.813E-26 | 0.15212381 | 1.2221E-21 |
| TNRC6C | 1.7845E-22 | -0.385644 | 3.7518E-18 |
| OSBPL5 | 1.3153E-21 | 0.89082815 | 2.7654E-17 |
| SORBS3 | 8.6704E-21 | -1.1081997 | 1.8229E-16 |
| NFE2 | 1.5675E-17 | 1.40243794 | 3.2956E-13 |
| WDR1 | 3.7433E-17 | 0.4283661 | 7.8699E-13 |
| CD151 | 6.9568E-17 | 0.90057166 | 1.4626E-12 |
| CELF2 | 1.3535E-16 | 0.2237476 | 2.8455E-12 |
| BST1 | 7.8714E-14 | 0.57138376 | 1.6549E-09 |
| PLEKHB1 | 9.5491E-14 | -1.1626737 | 2.0076E-09 |
| GRK2 | 4.2075E-13 | 0.30731171 | 8.8459E-09 |
| TTTY14 | 7.5552E-11 | -5.69154 | 1.5884E-06 |
| ADCY7 | 3.5461E-10 | 0.44533954 | 7.4552E-06 |
| SPNS3 | 8.7038E-10 | -0.361992 | 1.8299E-05 |
| NADK | 3.3517E-09 | 0.50946744 | 7.0466E-05 |
| ANKRD36C | 9.5222E-09 | -0.223823 | 0.00020019 |
| PTPN1 | 1.2269E-08 | -0.1603152 | 0.00025795 |
| CCDC28B | 2.143E-08 | 0.73500582 | 0.00045054 |
| ARAP1 | 3.7861E-08 | 0.48682279 | 0.00079599 |
| ARHGDIA | 6.7579E-08 | 0.19384423 | 0.00142078 |
| NEDD4L | 7.9801E-08 | 1.0356243 | 0.00167773 |
| GPR65 | 1.8549E-07 | 0.3880702 | 0.00389979 |
| MBOAT2 | 3.7958E-07 | 1.13383029 | 0.00798027 |
| ZNF138 | 4.7981E-07 | -0.2087157 | 0.01008762 |
| PARD3 | 7.6548E-07 | 2.72672057 | 0.01609346 |
| NT5C | 8.6231E-07 | 0.32832974 | 0.01812918 |
| P4HB | 1.4711E-06 | 0.22717087 | 0.03092934 |
| MRAS | 2.1238E-06 | 0.88183362 | 0.04465146 |
| KCNAB2 | 3.8327E-06 | 0.3109838 | 0.08057818 |
| WDR86 | 4.5999E-06 | -1.115558 | 0.09670785 |
| CBX4 | 1.1642E-05 | 0.31226661 | 0.24475823 |
| DTX1 | 1.466E-05 | -1.6987317 | 0.30820605 |
| GUK1 | 2.9006E-05 | 0.12980671 | 0.60982211 |
| SEMA6B | 3.847E-05 | 1.00698667 | 0.80878717 |
| PISD | 4.5397E-05 | 0.46863157 | 0.95443033 |
| DENND1A | 0.00013618 | 0.51868396 | 1 |
| EXOC3 | 0.00023719 | 0.43104327 | 1 |
| GTF3C1 | 0.00031484 | 0.40890099 | 1 |
| LMNA | 0.00056028 | 0.47966578 | 1 |
| PPP1R9B | 0.00064427 | 0.35310122 | 1 |
| GPR132 | 0.00068223 | -0.1528537 | 1 |
| HYAL2 | 0.00074974 | 0.65467075 | 1 |
| RASGRP2 | 0.00100323 | 0.16227666 | 1 |
| DGKD | 0.00146828 | 0.34040204 | 1 |
| SHANK3 | 0.00155998 | 5.41686917 | 1 |
| LENG8 | 0.00227226 | 0.26004437 | 1 |
| WBP2 | 0.00242743 | 0.46447189 | 1 |
| TEPP | 0.00389329 | -0.4899012 | 1 |
| NLGN2 | 0.00432331 | -1.8907332 | 1 |
| HPSE | 0.00439071 | 0.56786957 | 1 |
| HIBADH | 0.00575889 | -0.126269 | 1 |
| HSD3B7 | 0.00632598 | 1.02041042 | 1 |
| LAS1L | 0.00650545 | -0.1472979 | 1 |
| POU2F2 | 0.00726517 | 0.23922572 | 1 |
| ELF4 | 0.00982293 | 0.45209331 | 1 |
| MYO1C | 0.00997489 | -0.1585569 | 1 |
| BAHCC1 | 0.01119602 | -0.6500446 | 1 |
| TNFRSF8 | 0.01272649 | 0.59445181 | 1 |
| TRPC4AP | 0.01390717 | 0.2760527 | 1 |
| ATP5PF | 0.01714015 | 0.11857766 | 1 |
| UCP2 | 0.01797719 | 0.10733291 | 1 |
| FLII | 0.02400706 | 0.26439377 | 1 |
| TELO2 | 0.02763497 | 0.48088421 | 1 |
| SMG7 | 0.02900953 | 0.348811 | 1 |
| CLCN6 | 0.03023797 | 0.36168853 | 1 |
| CPQ | 0.03042593 | 0.22141296 | 1 |
| MARK4 | 0.03244993 | 0.36275969 | 1 |
| PPP1R12B | 0.03394383 | 0.37761721 | 1 |
| MAFK | 0.03813168 | 0.36698547 | 1 |
| RILP | 0.03864559 | 0.5521637 | 1 |
| PIK3R2 | 0.04018219 | -0.2505443 | 1 |
| TAF10 | 0.04467316 | 0.16035035 | 1 |
| UNKL | 0.04916106 | 0.40664105 | 1 |
| RTN4RL1 | 0.05127582 | 2.39879738 | 1 |
| ERV3-1 | 0.05300009 | 0.40470281 | 1 |
| SGPL1 | 0.05636502 | 0.29761609 | 1 |
| PRSS36 | 0.05882 | 0.74809992 | 1 |
| JMJD1C | 0.06014741 | 0.18741227 | 1 |
| ZMYND8 | 0.0664377 | 0.26737657 | 1 |
| EGFL8 | 0.07177457 | -0.8465905 | 1 |
| DNM1 | 0.07470833 | 0.87509832 | 1 |
| SMAD2 | 0.08418783 | 0.24562228 | 1 |
| MAFG | 0.09410829 | 0.40530104 | 1 |
| VASN | 0.10706928 | -1.7611163 | 1 |
| IGSF11 | 0.1101997 | -1.8530643 | 1 |
| DGKG | 0.11275204 | 0.54757416 | 1 |
| CHADL | 0.12147317 | 1.33139768 | 1 |
| WWP2 | 0.12442281 | 0.27546329 | 1 |
| ALKBH6 | 0.13022583 | 0.31019262 | 1 |
| RHOBTB2 | 0.13657697 | 0.45622729 | 1 |
| DND1 | 0.13843738 | -0.1080364 | 1 |
| SPEN | 0.15413431 | 0.19867335 | 1 |
| LUC7L3 | 0.16761809 | 0.10531822 | 1 |
| PRKD2 | 0.18156045 | 0.12022393 | 1 |
| UBL4A | 0.18382733 | 0.36204798 | 1 |
| INF2 | 0.18534652 | 0.44340752 | 1 |
| FKBP6 | 0.1884028 | 0.87762857 | 1 |
| ZNF280B | 0.19611672 | -0.1911178 | 1 |
| PUDP | 0.20809761 | 0.35440405 | 1 |
| NRXN2 | 0.21817354 | 0.60108785 | 1 |
| CDKN1A | 0.22115999 | 0.27119828 | 1 |
| EVPL | 0.23475548 | -1.2500877 | 1 |
| ATF6B | 0.2360908 | 0.20252168 | 1 |
| PRICKLE1 | 0.24412309 | -0.3719664 | 1 |
| DNAJC24 | 0.24515504 | 0.10476997 | 1 |
| PHIP | 0.24768034 | 0.1364582 | 1 |
| MYO1E | 0.25020171 | 0.5180485 | 1 |
| KCP | 0.26172521 | 0.96383934 | 1 |
| MCM3AP | 0.26445707 | 0.10514103 | 1 |
| CLUH | 0.2689713 | 0.14852369 | 1 |
| GRIK5 | 0.27654149 | -0.5423325 | 1 |
| B3GALT4 | 0.28308979 | 0.28823823 | 1 |
| SLC12A3 | 0.29436682 | 1.56612057 | 1 |
| PTBP1 | 0.30674249 | 0.18213258 | 1 |
| ATG12 | 0.32274557 | 0.10147617 | 1 |
| AKT1 | 0.32900699 | 0.19478488 | 1 |
| NFIC | 0.33270256 | 0.22944305 | 1 |
| AKR1A1 | 0.33348295 | 0.14907235 | 1 |
| LIMCH1 | 0.33446281 | 0.84631177 | 1 |
| CLPB | 0.33556487 | 0.28150937 | 1 |
| NCOR2 | 0.3480411 | 0.19870177 | 1 |
| RAPGEF5 | 0.35206961 | 1.3015113 | 1 |
| ASPSCR1 | 0.35504344 | 0.27172849 | 1 |
| THBS3 | 0.36332878 | 0.53219499 | 1 |
| NTM | 0.36530003 | -0.4046066 | 1 |
| RFTN1 | 0.3748112 | 0.24414551 | 1 |
| COL5A1 | 0.38500862 | -0.4602277 | 1 |
| TRIP13 | 0.38656184 | 0.63912889 | 1 |
| CNDP2 | 0.38758495 | 0.20080031 | 1 |
| EIF3B | 0.39221323 | 0.15321233 | 1 |
| CEP250 | 0.40145213 | 0.17769913 | 1 |
| MBD3 | 0.41019776 | 0.11358103 | 1 |
| MYB | 0.41569732 | -0.2426497 | 1 |
| DAXX | 0.41831975 | 0.17493688 | 1 |
| INTS3 | 0.42035205 | 0.157352 | 1 |
| CYP7B1 | 0.4416957 | 0.59740093 | 1 |
| PLPPR3 | 0.4416957 | 0.57450771 | 1 |
| SLC1A7 | 0.44470119 | 0.40904473 | 1 |
| TBC1D16 | 0.46727765 | 0.56872794 | 1 |
| KIF7 | 0.46955828 | 1.00078735 | 1 |
| STK25 | 0.48373683 | 0.15962207 | 1 |
| PRKCH | 0.4889199 | 0.22253146 | 1 |
| FAM131B | 0.49449892 | 0.87596914 | 1 |
| RBM4 | 0.51292578 | 0.17338381 | 1 |
| MRPL55 | 0.51423069 | 0.17165456 | 1 |
| SLC4A1 | 0.52210956 | 0.37962174 | 1 |
| DDIT4 | 0.5255194 | 0.25928567 | 1 |
| TOP3B | 0.54301125 | 0.23596435 | 1 |
| GCNT2 | 0.54351283 | 0.1476114 | 1 |
| EGLN1 | 0.54660789 | 0.15717289 | 1 |
| MAGED2 | 0.55261631 | 0.36138655 | 1 |
| SCN3A | 0.55361833 | -0.2474617 | 1 |
| NUDT19 | 0.55387527 | 0.10446377 | 1 |
| DDX39B | 0.56069423 | 0.1424662 | 1 |
| ZNF521 | 0.5683875 | 0.31927977 | 1 |
| ELF5 | 0.57241538 | 1.1941904 | 1 |
| SMAD9 | 0.57241538 | 0.69995733 | 1 |
| KDM5C | 0.57648023 | 0.24536623 | 1 |
| AIF1L | 0.60124204 | 1.50229509 | 1 |
| TMEM117 | 0.61612072 | 0.15665531 | 1 |
| TREX1 | 0.63331142 | 2.38450491 | 1 |
| ABR | 0.63783537 | 0.20451921 | 1 |
| GPT2 | 0.63849557 | 0.58121281 | 1 |
| BEND7 | 0.64122806 | 0.80464477 | 1 |
| FBRSL1 | 0.64765866 | 0.25325121 | 1 |
| HEXDC | 0.64973207 | 0.16881312 | 1 |
| SORCS2 | 0.65667039 | 1.11104144 | 1 |
| CPT1A | 0.656869 | 0.18235934 | 1 |
| SPATC1 | 0.66055228 | 1.85807874 | 1 |
| KHDRBS3 | 0.66474969 | 0.48323441 | 1 |
| RNF138 | 0.67534051 | 0.10568838 | 1 |
| TRAPPC9 | 0.6788147 | 0.19774944 | 1 |
| HDAC4 | 0.70272336 | 0.21947724 | 1 |
| PHRF1 | 0.70478571 | 0.22356324 | 1 |
| WDR83 | 0.72135212 | 0.16900056 | 1 |
| TNS2 | 0.72725362 | 0.35483537 | 1 |
| RECQL5 | 0.73032374 | 0.34405611 | 1 |
| MYO18A | 0.73761798 | 0.12617548 | 1 |
| TMOD1 | 0.74228502 | 0.4726498 | 1 |
| TMEM184A | 0.74882955 | 0.1838326 | 1 |
| HGS | 0.76349371 | 0.18793453 | 1 |
| BRF1 | 0.77887299 | 0.19857188 | 1 |
| CLCN2 | 0.80824001 | 0.17572107 | 1 |
| PQLC2 | 0.82716487 | 0.34846378 | 1 |
| SBNO2 | 0.83174852 | 0.19126618 | 1 |
| SLC50A1 | 0.8384256 | 0.21013086 | 1 |
| ADGRB2 | 0.83896777 | 0.15852844 | 1 |
| MAP1LC3A | 0.84106324 | 0.26534844 | 1 |
| PPP2R1A | 0.86700346 | 0.16660306 | 1 |
| CCDC88C | 0.87343712 | 0.12026194 | 1 |
| OXSM | 0.87695275 | 0.2056132 | 1 |
| CCDC115 | 0.88794104 | 0.12437478 | 1 |
| PPP2R2B | 0.89015365 | 0.17691776 | 1 |
| ALG3 | 0.89254186 | 0.16384232 | 1 |
| BEST4 | 0.89263031 | 0.20348477 | 1 |
| UPF1 | 0.90109012 | 0.17642776 | 1 |
| PRKX | 0.90287596 | 0.17129548 | 1 |
| PAFAH1B1 | 0.90652778 | 0.15463118 | 1 |
| COL1A1 | 0.92574055 | 0.4216665 | 1 |
| STK11 | 0.9359116 | 0.16917784 | 1 |
| CUL9 | 0.95646308 | 0.2803582 | 1 |
| TNXB | 0.95997365 | 0.96262291 | 1 |
| ZBTB12 | 0.96429923 | 0.90480128 | 1 |
| UEVLD | 0.97010254 | 0.14585561 | 1 |
| LRRC32 | 0.98095647 | 1.24090094 | 1 |
| CPT1B | 0.99617976 | 0.32955499 | 1 |
| ARFGEF3 | 1 | 4.4572629 | 1 |

*** adjusted by Bonferroni correction**

**Supplemental references**

1. Nauffal V. Di Achille P. Klarqvist MDR. Cunningham JW. Hill MC. Pirruccello JP. Weng LC. Morrill VN. Choi SH. Khurshid S. Friedman SF. Nekoui M. Roselli C. Ng K. Philippakis AA. Batra P. Ellinor PT. Lubitz SA. Genetics of myocardial interstitial fibrosis in the human heart and association with disease. *Nat Genet* 2023; **55**:777-786.
